# Log Mean Divisia Index Decomposition Analysis of the Demand for Building Materials: Application to Concrete, Dwellings, and the U.K.

**DOI:** 10.1021/acs.est.0c02387

**Published:** 2021-02-20

**Authors:** He He, Rupert J. Myers

**Affiliations:** † Current address: Department of Civil and Environmental Engineering, Imperial College London, Skempton Building, London, SW7 2AZ, United Kingdom; ‡ School of Engineering, The University of Edinburgh, King’s Buildings, Sanderson Building, Edinburgh, EH9 3FB, United Kingdom

## Abstract

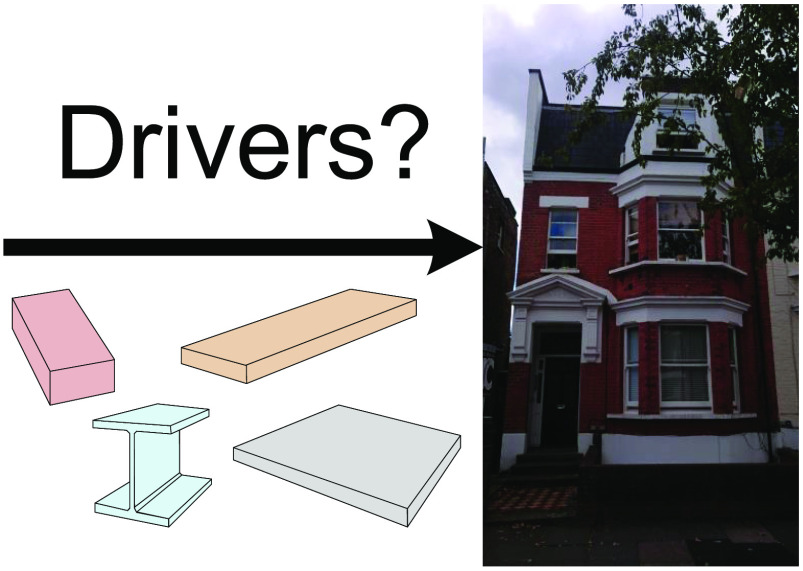

Dwellings are material intensive products. To date, material use in dwellings has been investigated mainly using economic (exogenous) or dwelling (endogenous) drivers, with few studies comprehensively combining both. For the first time, we identify a comprehensive set of such drivers of demand for building materials and analyze them using the logarithmic mean divisia index (LMDI) method. We combine the LMDI method, the concept of dynamic material flow analysis, and physical and monetary flows to decompose the demand for building materials into the following six effects: material intensity, floor area shape, dwelling type, dwelling intensity, economic output, and population. We analyze these six effects on demand for concrete in new dwellings in the U.K. from 1951 to 2014, classified into six dwelling types and four subregions. Of these six effects, the material intensity effect is the most important, overall contributing to increasing concrete demand by +79 Mt from 1950 to 2014, while the dwelling intensity effect plays an opposite role, overall reducing concrete demand from 1950 to 2014 by −56 Mt. The economic output effect is also significant (+38 Mt from 1950 to 2014). A comparative analysis of the six effects in the four U.K. nations reveals that most of the effects arise from England, while the other nations have minor effects due to their smaller populations. Our results show that changes to the demand for concrete in the U.K. fluctuate and have mainly remained between ±30 Mt year^–2^ from 1950 to 2014, and thus the inflows of concrete into the in-use stock of dwellings have experienced neither entirely increasing or decreasing trends during this period. This study contributes to understanding changes in resource demand due to social, economic, and technological factors and thus improves the capability to reliably and quantitatively model the use of materials in the built environment.

## 1 Introduction

Residential buildings (hereafter dwellings) are essential to the provision of shelter. By 2050, at least 20 billion m^2^ of additional floor area will be needed to provide shelter for 9.7 billion people, up from 235 billion m^2^ in 2016.^1^ They are also responsible for at least 19% of energy-related CO_2_ emissions,^1^ of which an increasing fraction will be associated with the embodied component of their life cycles as operational CO_2_ emissions reduce.^2,3^ The growing understanding of the importance of material efficiency in climate change mitigation has further increased attention toward CO_2_ emissions associated with building materials.^4−7^

The most common building materials include concrete, steel, bricks, timber, and glass.^8,9^ Their raw materials, e.g., limestone, gravel, iron ore, clay, wood, and sand, tend to be abundant on the global level and relatively inexpensive.^10−12^ However, construction minerals such as quarried natural aggregates (e.g., gravel, sand) may have local availability constraints.^13^ Constructing new dwellings and maintaining existing dwellings uses a huge amount of buildings materials.^14,15^ For example, it is reported that the total stock of building materials in China will increase to 235.7 Gt in 2050.^16^ Cement production in China was 2.20 Gt in 2019^17^ and is expected to be at or near its peak due significantly to new dwelling construction.^18^

Demand for building materials can be modified through various measures, e.g., lifetime extension,^19^ material substitution,^20^ lightweight design,^21^ and re/upcycling.^22^ Overall, these measures may significantly modify the amounts and types of materials used, as well as reduce overall life cycle CO_2_ emissions associated with buildings and infrastructure.^23^

The stock-flow dynamics of building materials and their associated environmental impacts are commonly analyzed using dynamic material flow analysis (dMFA).^18^ This method uses mass conservation to quantify physically consistent demand, use, and disposal of materials or products in space and time.^24,25^ It has been applied to quantify the stock-flow dynamics of dwellings^26^ and nonresidential buildings^27^ and building materials such as steel,^28^ wood,^29^ and cement.^30^ Applications of dMFA to buildings on the national scale are also common, including The Netherlands,^31^ Norway,^32^ and China.^16,33^ The dMFA method has also been used to investigate end-of-life material flows and their potential utilization as secondary resources in Hong Kong up to the year 2050.^34^ Although the combination of MFA and geographic information systems has been used to illustrate the detailed accumulation of construction materials in Bejing’s buildings and infrastructure in 2018, the lack of temporal dynamics in that study hindered the application of its results to policy design.^35^ These approaches have however been more comprehensively combined. For example, a dMFA model using bottom-up building stock data combined with three-dimensional and geo-referenced building data was developed to calculate material stocks in Swiss residential buildings in high detail and used multiple scenarios to investigate potential changes of the Swiss building stock up to 2050.^36^ MFA has also been applied to explore the relationship between material consumption and economic growth in an Irish city region during the period 1992–2002.^37^ Key factors influencing the metabolism of a household have been analyzed using MFA, showing that the material inputs and stocks depend on household size and income.^38^ The stock dynamics of an urban environment have also been assessed using dMFA, which illustrates the drivers (e.g., population, building types, and materials) of the building stock at material, structure, and building type levels.^39^ In summary, dMFA has used population as a key socio-economic driver, and often additionally building type and material intensity, to quantify material stocks and flows.

Other socio-economic drivers, including fixed investment and gross domestic product (GDP),^40−42^ have also been used to analyze demand for building materials. These socio-economic drivers are usually studied using *IPAT* type decomposition analyses,^43,44^ where *I* is an environmental indicator (e.g., energy consumption), *P* is population (e.g., persons), *A* is affluence (e.g., GDP per capita), and *T* is a technology term (e.g., energy consumption per unit of GDP in the region).^45,46^ For example, the U.K. population increased from ∼49 million in 1945 to ∼66 million in 2018;^47^ the U.K. gross domestic product (GDP) increased from ∼£9.6 billion in 1945 to £2,144 billion in 2018,^48^ and the contribution of the construction sector to GDP in the U.K. was ∼£117 billion in 2018 (6% of the total).^49^

However, demand for building materials may also change as a function of user preferences: e.g., the proportion of detached homes used in Scotland increased from 5% (on average) between 1965 to 1982, to 10% after 1982.^50^ Such user preferences drive demand for building materials and so should be considered in studies analyzing them. As endogenous drivers of the demand of building materials, building typologies and floor area per capita are not only affected by economic development^51,52^ but also by other factors, such as behaviors of dwellers^53^ and building codes.^54^

Relatively few studies have comprehensively analyzed drivers of demand for building materials. *IPAT* type decomposition analysis has been used to study the aggregate “material stock” of urban and rural regions in Japan by disaggregating gross prefectural product into the primary (e.g., fishing and mining), secondary (e.g., manufacturing), and tertiary (e.g., education and finance services) sectors.^55^ The Kaya identity, which has a similar form to the *IPAT* equation but includes an emissions intensity term, has also been used to examine building stock improvement measures.^35,56^ A critical review has aggregated “A” for GDP per capita and “B” for floor area per GDP into the *IPAT* method for the analysis of dynamics of construction materials in urban building stocks.^56^ Although this application and review of the *IPAT* decomposition method covered major socio-economic drivers of the demand for building materials stock, they do not focus on explaining the inflow dynamics of building materials.

Drivers of physical flows can also be analyzed using index decomposition analysis (IDA), which is a method that can be used to analyze contributions of different drivers to changes in commodity production.^57^ This method has previously been used to assess the absolute and relative importance of different drivers in explaining energy and carbon emissions temporally and spatially^58^ and has advantages in analyzing panel data, which are multidimensional data involving measurements over time.^59−61^ Time-series data can be considered as a special case of panel data that are in one dimension. The logarithmic mean divisia index (LMDI) method is currently considered to be the most popular IDA approach.^59^ It has been widely used to assess the effects of socio-economic drivers (population, investment, and per capita GDP) and characteristics of various industrial sectors (food industry, iron and steel industry, and electricity) on energy, CO_2_ emissions, food consumption, water use, and material resources.^60,62−64^ For example, the LMDI method has been used to analyze the drivers of material consumption globally from 1995 to 2008.^65^ Drivers of the agricultural water footprint have been analyzed using the LMDI method; this study constructed agricultural water efficiency as the ratio of the amount of water to gross value added (GVA) of the agricultural sector.^66^ Drivers of metal stocks across 10 Chinese megacities have been analyzed through the combination of *IPAT* and LMDI methods from 1980 to 2016.^35^ However, this combination of methods has not yet been specifically applied to investigate the drivers of demand for building materials, including different building types and regional differences.

LMDI decompositions can be classed as multiplicative or additive. Multiplicative decomposition is used to analyze ratios of change in effects from year to year, whereas additive decomposition is used to analyze differences in the amounts of change in effects from year to year.^59^ Here, we decompose changes in demand for building materials in the U.K. using the additive LMDI method, focusing on concrete and new dwellings to illustrate our approach. Our overall aims are to quantitatively determine the importance of effects on demand for concrete in the U.K. and to understand how these contributions have changed over time (1951–2014). This information is directly relevant for flow-driven dMFA studies and can be indirectly used to analyze material stocks and environmental impacts. We clarify the effects of regional differences on demand for concrete by disaggregating our data and analysis into four U.K. subregions and discuss these results in light of building material use more generally.

## 2 Methodology

### 2.1 Modeling Approach

**Figure 1:**
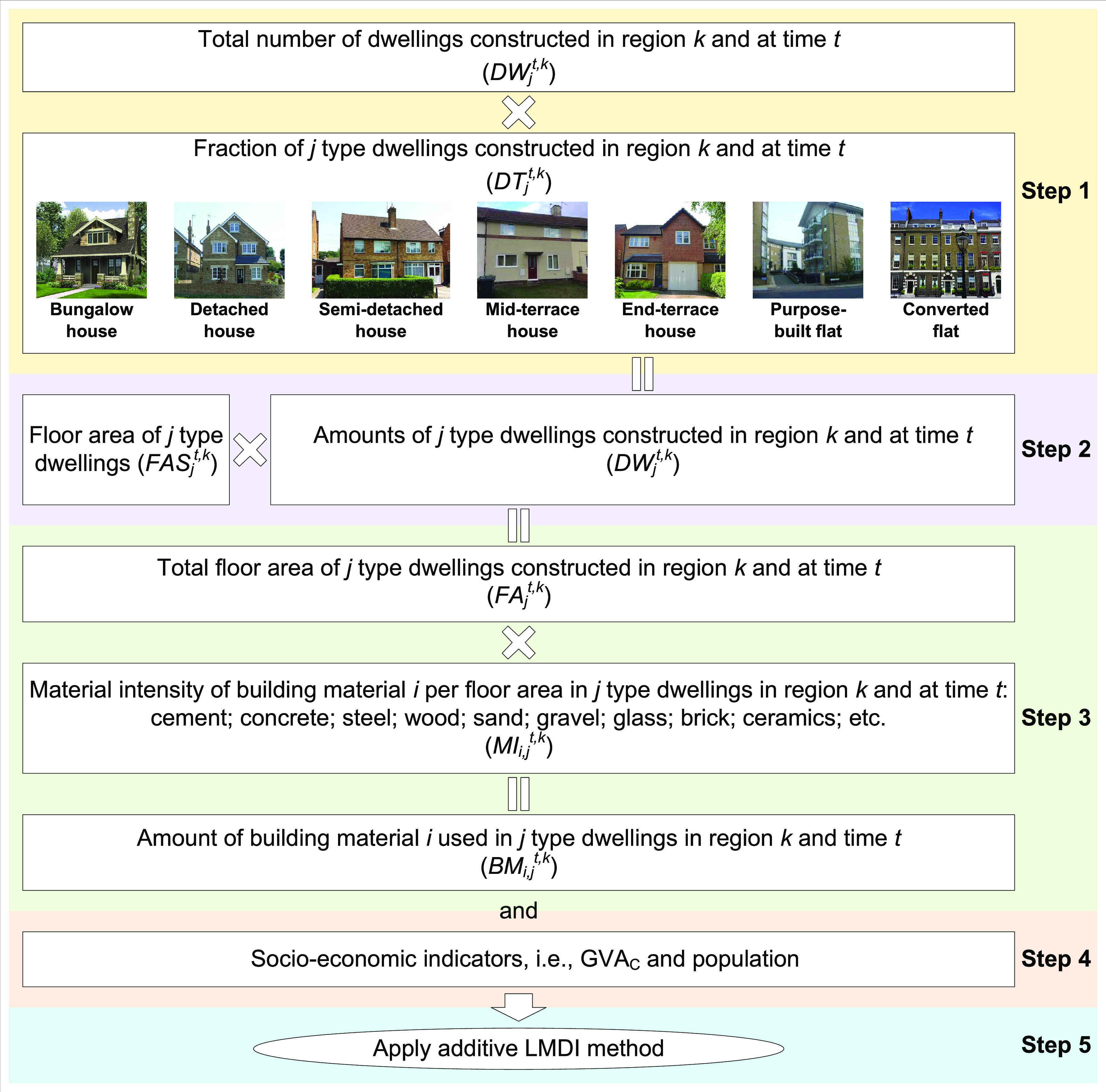
LMDI method developed and applied here to analyze demand for concrete in new dwellings in the U.K. from 1951 to 2014. The parameters shown, defined in terms of construction occurring in region *k* and at time *t*, are DW^*t*,*k*^, total amount of new dwellings; DT_*j*_^*t*,*k*^, the fraction of *j* type dwellings; DW_*j*_^*t*,*k*^, amount of *j* type dwellings; FAS_*j*_^*t*,*k*^, floor area of *j* type dwellings; FA_*j*_^*t*,*k*^, total floor area of *j* type dwellings, MI_*i*,*j*_^*t*,*k*^, material intensity of building material *i* per floor area in *j* type dwelling; BM_*i*,*j*_^*t*,*k*^, amount of building material *i* used in *j* type dwelling.

The method described here can be applied to analyze materials in general. Therefore, we develop it here referring to “building materials” and focus our subsequent analysis on concrete due to the massive scale in which it is used. It includes the following five steps (Figure 1): (1) Calculate the amounts of dwellings constructed in different time periods and regions, classified by type. (2) Estimate the floor areas of different types of dwellings in different time periods and regions. (3) Calculate the amount of building materials used in different types of new dwellings in different time periods and regions. (4) Evaluate the socio-economic indicators (e.g., population and gross value added of the construction sector (GVA_C_)) in different time periods and regions. And (5), define the demand for building materials based on the exogenous (socio-economic indicators) and endogenous (concrete intensity, floor area of new dwellings, types of new dwellings) drivers using IDA, and apply the LMDI method to analyze their effects on demand for building materials.

The calculation details from step 1 to 4 are shown in Supporting Information S1 (Appendix S1). In step five, we establish an equation relating changes in demand for building materials in new dwellings, and its drivers, based on an extension of the IDA method.^61^ The basic equation describing the IDA method can be written as follows (eq 1):
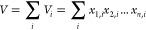
1where the aggregate indicator *V* is disaggregated into *i* subcategories and *n* factors (i.e., *x*_1,*i*_, *x*_2,*i*_, ..., *x*_*n*,*i*_) contribute to its changes during the time period of analysis.^59^ Here, we use *V* to describe the demand for building materials in new dwellings constructed in the U.K.

Assuming that there are *j* types of dwellings, the demand for building material *i* across all types of dwellings in the U.K. at time *t* can be expressed as (eq 2):
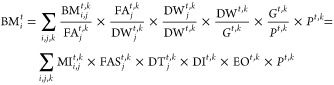
2where BM_*i*_^*t*^ (Mt year^–1^) is the total demand of building material *i* across all types of dwellings at time *t*, *G*^*t*,*k*^ (£ million) is the GVA_C_ in region *k* at time *t*, and *P*^*t*,*k*^ (persons) is the population in region *k* at time *t*. The summation of GVA of all sectors is calculated by the GDP with subsidies added and taxes deducted.^67^ The GVA can better explain economic activities with the effect of subsidies and without the effect of taxes (tariffs) on products. The GVA can be used for measuring the contribution to GDP made by an individual producer, industry, or sector.^67^ For example, economic activities that are characteristic of tourism are assessed through the GVA of the tourism sector.^68^ Costs in a carbon leakage assessment are calculated for each sector as a percentage fraction of the GVA of each sector.^69^ Here, we assess the effect of construction sector economic activity on the demand for building materials, specifically concrete.

Table 1 shows the parameters, units, and descriptions of variables used in eq 2 and in the main text.

**Table 1:** Definitions of Variables Used in eq 2 and in the Main Text

variable	parameter	unit	description
BM_*i*_^*t*,*k*^		Mt year^–1^	total amount of building material *i* demanded in region *k* and at time *t*
BM_*i*,*j*_^*t*,*k*^		Mt year^–1^	amount of building material *i* demanded in dwelling type *j* in region *k* and at time *t*
FA_*j*_^*t*,*k*^		m^2^ year^–1^	total floor area of type of dwellings *j* demanded in region *k* and at time *t*
DW_*j*_^*t*,*k*^		–	amount of type of dwellings *j* demanded in region *k* and at time *t*
DW^*t*,*k*^		dwellings year^–1^	total amount of dwellings demanded in region *k* and at time *t*
*G*^*t*,*k*^		£million	GVA of the construction sector (GVA_C_) in region *k* and at time *t*
*P*^*t*,*k*^		persons	population of region *k* at time *t*
MI_*i*,*j*_^*t*,*k*^	BM_*i*,*j*_^*t*,*k*^/FA_*j*_^*t*,*k*^	Mt m^–2^	intensity of building material *i* in type of dwellings *j* demanded in region *k* and at time *t*
FAS_*j*_^*t*,*k*^	FA_*j*_^*t*,*k*^/DW*_j_^t^*^,^*^k^*	m^2^ dwellings^–1^	floor area in type of dwellings *j* demanded in region *k* and at time *t*
DT_*j*_^*t*,*k*^	DW*_j_^t^*^,^*^k^*/DW*^t^*^,^*^k^*	–	proportion of the amount of type of dwellings *j* demanded to the total amount of dwellings demanded in region *k* and at time *t*
DI^*t*,*k*^	DW^*t*,*k*^/*G^t^*^,^*^k^*	dwellings year^–1^ £million^–1^	proportion of the total amount of dwellings demanded relative to the GVA_C_ in region *k* and at time *t*
EO^*t*,*k*^	*G^t^*^,^*^k^*/*P*^*t*,*k*^	£million persons^–1^	proportion of the GVA_C_ to the population (GVA_C_ per capita) in region *k* and at time *t*

We use the additive LMDI method here to analyze changes in demand for each building material *i* (ΔBM_*i*_^*k*^, Mt year^–2^) in yearly time steps (*T* to *T* + 1) over the period 1951 to 2014, as shown in eq 3. Here, BM_*i*_^*t*,*k*^ represents the inflows of building material *i* into the in-use stock at time *t*, and ΔBM_*i*_^*k*^ represents the rate of change of inflows of building material *i* into the in-use stock between times *t* and *t* + 1 (unit time steps; we use yearly time steps here) in region *k*:
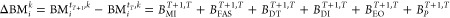
3In eq 3, the effects *B*_MI_^*T*+1,*T*^, *B*_FAS_^*T*+1,*T*^, *B*_DT_^*T*+1,*T*^, *B*_DI_^*T*+1,*T*^, *B*_EO_^*T*+1,*T*^, and *B*_*P*_^*T*+1,*T*^ describe changes in demand for building material *i* from year *T* to *T* + 1 and are defined as follows (Table 2): *B*_MI_^*T*+1,*T*^ (Mt year^–2^) is the material intensity effect, which relates changes in material intensity to the change in demand for building material *i*. *B*_FAS_^*T*+1,*T*^ (Mt year^–2^) is the floor area shape effect, which relates changes in characteristic floor area of different dwelling types to the change in demand for building material *i*. *B*_DT_^*T*+1,*T*^ (Mt year^–2^) is the dwelling type effect, which relates changes in the relative amount of each dwelling type demanded (with respect to all new dwellings) to the change in demand for building material *i*. *B*_DI_^*T*+1,*T*^ (Mt year^–2^) is the dwelling intensity effect, which relates changes in the number of new dwellings per GVA_C_ to the change in demand for building material *i*. *B*_EO_^*T*+1,*T*^ (Mt year^–2^) is the economic output effect, which relates changes in economic output (GVA_C_ per capita) to the change in demand for building material *i*. And, *B*_*P*_^*T*+1,*T*^ (Mt year^–2^) is the population effect, which relates changes in population to the change in demand for building material *i*.

**Table 2:** Definitions of the Effects in eq 3 and Used Here to Quantify Changes in Demand for Building Materials in the U.K. between 1951 and 2014

effect	definition^a^
*B*_MI_^*T*+1,*T*^	
*B*_FAS_^*T*+1,*T*^	
*B*_DT_^*T*+1,*T*^	
*B*_DI_^*T*+1,*T*^	
*B*_EO_^*T*+1,*T*^	
*B*_*P*_^*T*+1,*T*^	

a *L*(*x*, *y*) is the logarithmic average of two positive numbers and *y*, given by  for *x* ≠ *y*.

Therefore, our model describes the change in demand for a building material (*i*) as a function of changes in material intensity (MI), floor area shape (FAS), dwelling type (DT), dwelling intensity (DI), economic output of the construction sector (EO), and population (*P*). The mathematical definitions of these effects are shown in Table 2.

There are eight LMDI methods, which can be classified into three dimensions: by decomposition type (additive; multiplicative); by method, based on Vartia indices I and II (LMDI-I; LMDI-II); and by indicator type (quantity indicator, e.g., energy consumption; intensity indicator, e.g., energy consumption per value added).^59^ The additive decomposition type estimates absolute changes in material consumption, while the multiplicative decomposition type calculates relative changes.^70^ Here, we aim to estimate absolute changes in demand for concrete used in new dwellings. Therefore, we apply additive LMDI-I using a quantity indicator (ΔBM_*i*_^*k*^) to analyze the six temporal decomposition effects defined in eq 3 above (Table 2).

A negative value for an effect means that it induces a decrease in material demand, whereas a positive value for an effect means that it induces an increase in material demand. The magnitude of an effect indicates the extent to which material demand increases or decreases.

In summary, eq 2 shows how we decompose the demand for concrete used in dwellings in the U.K. and its four subregions (England, Scotland, Wales, Northern Ireland) during 1951–2014 into six drivers (based on the data sources described in section 2.2 below). These drivers are then used in eq 3 to quantify the effects of material intensity, floor area shape, dwelling type, dwelling intensity, economic output, and population on changes in demand for building material *i* in region *k* and at time *t*.

### 2.2 Data Sources

We collected data on the amounts of new dwellings (DW^*t*,*k*^) constructed annually in England, Scotland, Wales, and Northern Ireland from 1946 to 2018.^71−73^ For each of the four U.K. subregions, population and GVA data were obtained from the Office for National Statistics.^46,74−76^ Data gaps in the fraction (DT_*j*_^*t*,*k*^) and floor area (FAS_*j*_^*t*,*k*^) of *j* type dwellings in Wales and Northern Ireland, and GVA_C_ in the four subregions of the U.K., were estimated following the procedure described in Supporting Information S1 (Appendix S2). Reported GVA data include the effect of inflation and exclude taxes (less subsidies) on products; therefore, we deflated the reported GVA data before using them here. We applied the retail price index to deflate the GVA_C_ in the U.K. rather than the consumer price index because the former index includes more housing cost items than the latter.^75^ Deflation of the GVA_C_ aims to remove effects of price changes when considering changes in quantities between consecutive years.^77^ We use the deflated GVAc to produce all of our results here.

We analyzed this complete data set (including estimates to fill data gaps) to illustrate these socio-economic drivers and their trends (Supporting Information S1, Appendix S3). We also estimated concrete intensities in new U.K. dwellings; compared concrete intensities in these new U.K. dwellings with those in Norway, Sweden, and The Netherlands; and conducted a sensitivity analysis to quantify the uncertainty in our input data (Supporting Information S1, Appendix S4). Our study assumes that the concrete intensities of different types of new dwellings in Northern Ireland are the same as those in England, and concrete intensities of different types of new dwellings in Wales are the same as those in Scotland. This assumption does not significantly affect our results and is justified by the similar building regulations used in these countries.

## 3 Results and Discussion

### 3.1 Material Intensity Effect

**Figure 2:**
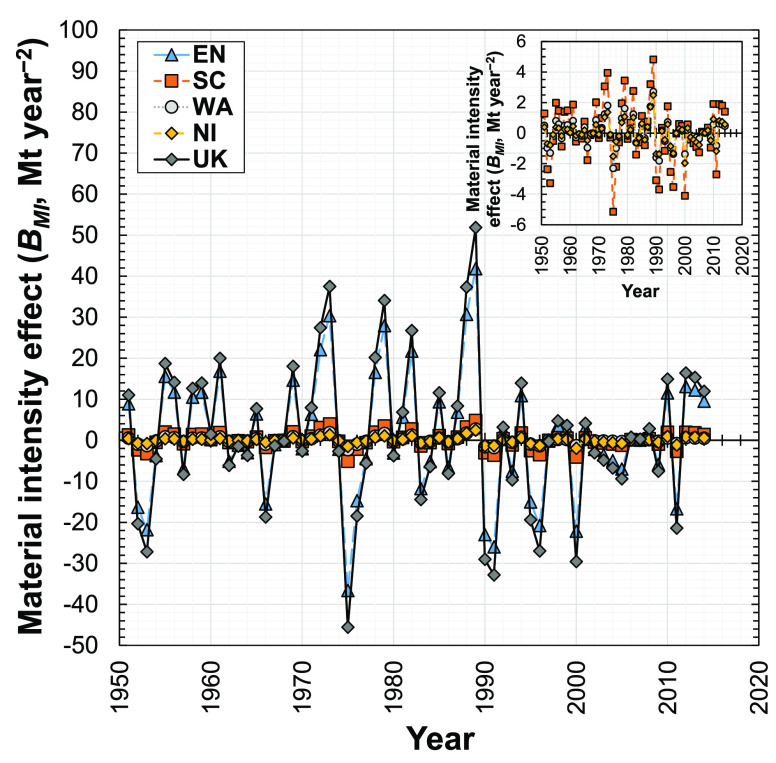
Influence of the material intensity effect on the change in demand for concrete in new dwellings in the U.K. and its four subregions (EN, England; SC, Scotland; WA, Wales; NI, Northern Ireland; U.K., United Kingdom) from 1951 to 2014.

Since our analysis focuses on concrete (= building material *i*), here the material intensity effect (*B*_MI_^*T*+1,*T*^) indicates the change in demand for concrete in new dwellings with respect to changes in the amount of concrete per floor area (i.e., material intensity). Figure 2 shows that the material intensity effect fluctuated in the U.K. and its subregions during 1951–2014. The material intensity effect in the U.K. has a maximum value of 51.8 Mt year^–2^ in 1989 and a minimum value of −45.6 Mt year^–2^ in 1975 (Figure 2). The estimated concrete intensity in new dwellings in the U.K. also fluctuated over this time period, from 1023 kg m^–2^ in 1951 to 1322 kg m^–2^ in 2014 and peaking at 2116 kg m^–2^ in 1989 (Supporting Information S1, Figure S3).

The material intensity effect in England fluctuated similarly to the U.K. trend, peaking at 41.8 Mt year^–2^ in 1989 and −36.6 Mt year^–2^ in 1975 (Figure 2). Scotland, Wales, and Northern Ireland follow similar trends, but all at significantly lower magnitudes: the material intensity effect induces less than ∼5 Mt year^–2^ change in demand for concrete in any individual year during the period of 1951–2014 for these three subregions. This explains why the U.K. and English material intensity effects are similar. These results indicate that a reduction in material intensity in new dwellings in England is needed to reduce the demand for concrete in the U.K. This may be achieved by using lightweight concrete and shape optimization of structural members in high-concrete dwelling types^78−80^ and substitution of concrete for other construction materials, e.g., timber.^81^ Multistory apartments (purpose-built flats) constructed by lightweight concrete and shape optimization, and prefabricated reusable modules, can reduce embodied environmental impacts of buildings.^82^

### 3.2 Floor Area Shape Effect

**Figure 3:**
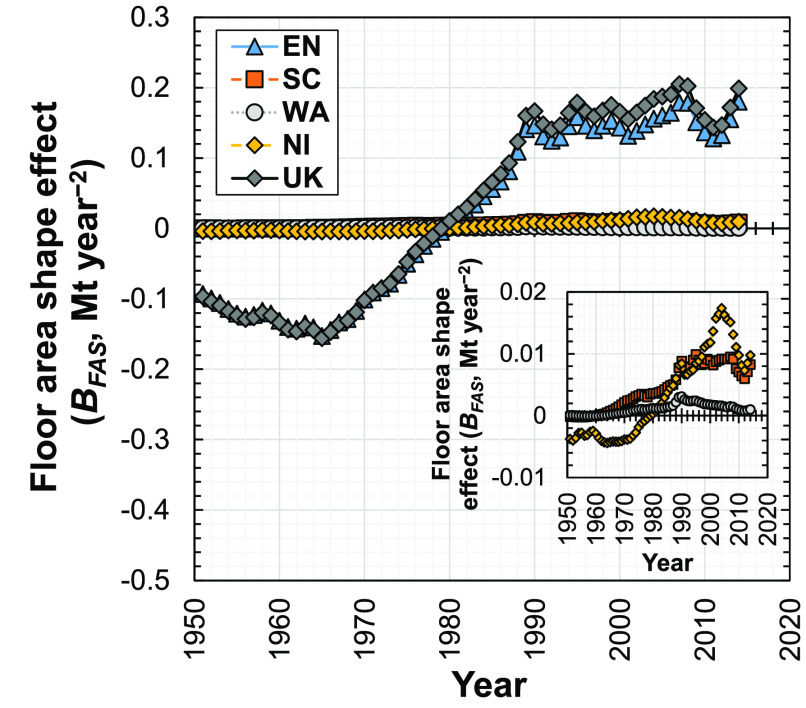
Influence of the floor area shape effect on the change in demand for concrete in new dwellings in the U.K. and its four subregions (EN, England; SC, Scotland; WA, Wales; NI, Northern Ireland; U.K., United Kingdom) from 1951 to 2014.

The floor area shape effect (*B*_FAS_^*T*+1,*T*^) for the U.K. was negative during 1951 to 1979 (Figure 3), indicating that during this period, changes in the floor area of new dwellings reduced concrete demand. However, from 1980 to 2014, the floor area shape effect for the U.K. was positive, inducing an increase in demand for concrete in new dwellings. The floor area shape effect decreased noticeably but remained positive between 2007 and 2011 and then more recently returned to its 2007 value in 2014. This decrease in floor area shape effect reflects the drop in number of new dwellings constructed between 2007 and 2010 (Figure S2d, Supporting Information S1), consequently reducing demand for concrete to smaller yet positive values.

The floor area shape effect is similar in England and the U.K.; its values for the three other subregions (Scotland, Wales, and Northern Ireland) are comparatively small. The floor area shape effects in the U.K. and its four subregions are all similar, which mostly peak at around 2007. We disaggregated the floor area shape effect in England into seven subeffects, representing the floor area shape effects for bungalow houses, detached houses, midterrace houses, purpose-built flats, end-terrace houses, semidetached houses, and converted flats, finding that the floor area shape subeffect for detached houses is the most important (Appendix S5, Supporting Information S1). However, the magnitudes of these floor area shape subeffects (on the order of ∼0.1 Mt year^–2^ or smaller) are significantly smaller than the magnitude of the material intensity effect (on the order of ∼10 Mt year^–2^, Figure 2), meaning that the latter effect is more important in changing the demand for concrete in new dwellings.

### 3.3 Dwelling Type Effect

**Figure 4:**
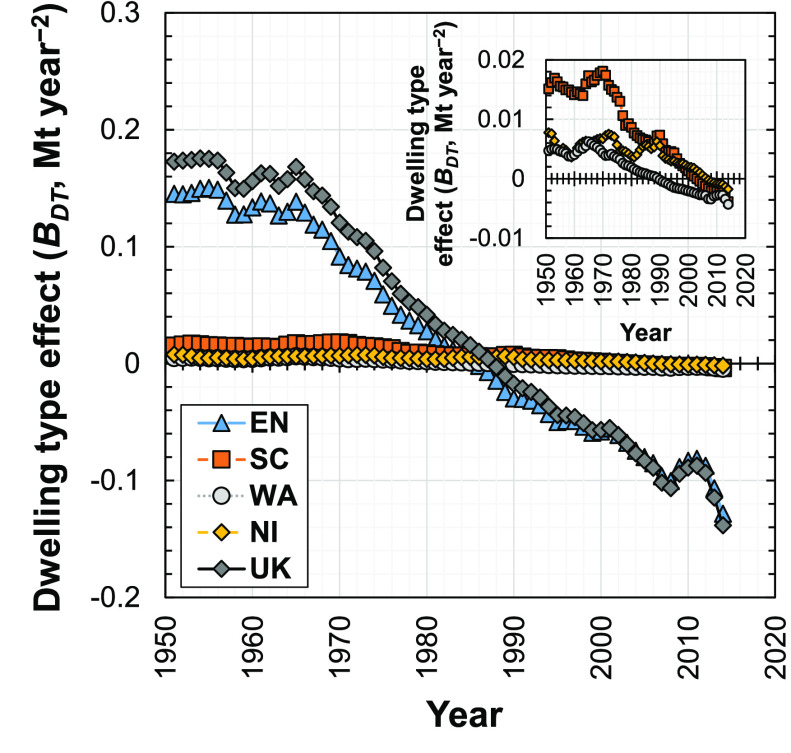
Influence of the dwelling type effect on the change in demand for concrete in new dwellings in the U.K. and its four subregions (EN, England; SC, Scotland; WA, Wales; NI, Northern Ireland; U.K., United Kingdom) from 1951 to 2014.

The dwelling type effect (*B*_DT_^*T*+1,*T*^) in the U.K. fluctuates from 1951 to 1965 but remains at an approximately constant positive value, with a maximum of 0.18 Mt year^–2^ in 1954 (Figure 4). The dwelling type effect in the U.K. generally experienced a decreasing trend from 1965 to 2014 and is negative from 1988 (Figure 4). Therefore, prior to 1988 the changes in types of new dwellings increased the demand for concrete in the U.K., with the opposite, i.e., decreasing demand for concrete, occurring after 1988.

Similar trends are observed for the dwelling type effect in the four U.K. subregions (Figure 4), with more positive values before the 1970s and less positive and eventually negative values thereafter. For example, in 2008, the dwelling type effect induced a reduction in the demand for concrete in England by 0.10 Mt year^–2^ and in Scotland by 0.002 Mt year^–2^. The dwelling type effect for England dominates the effects for the other three subregions and shows a similar trend to that of the U.K.

**Figure 5:**
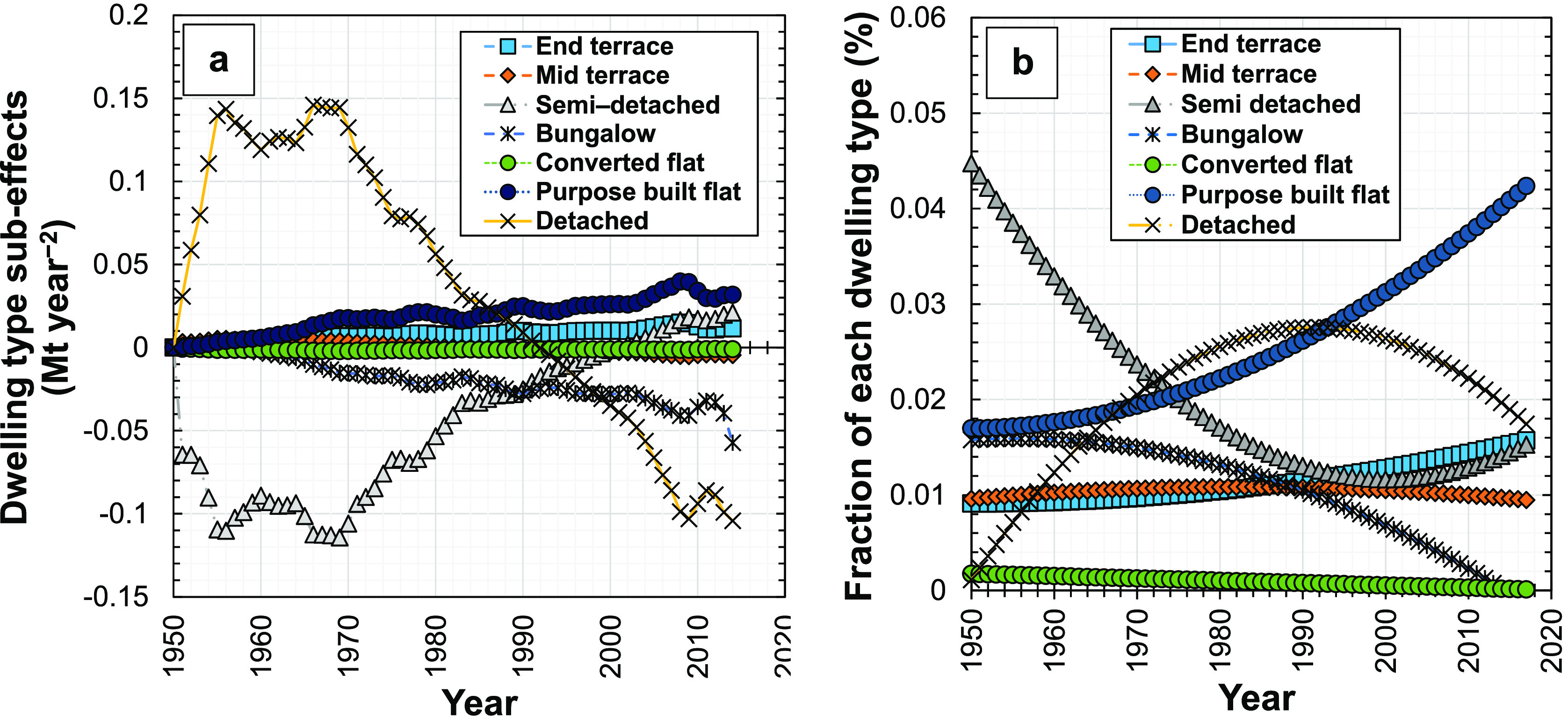
Results for the seven new dwelling types in England during 1951–2014: (a) influence of dwelling type subeffects on the change in demand for concrete in new dwellings and (b) the proportions of dwellings of different types relative to the total number of new dwellings.

We disaggregated the dwelling type effect in England into seven subeffects (Figure 5a), one for each of the seven dwelling types analyzed: bungalow houses, detached houses, midterrace houses, purpose-built flats, end-terrace houses, semidetached houses, and converted flats. The contribution from detached houses is the most significant, although the magnitudes of these dwelling type subeffects (on the order of ∼0.1 Mt year^–2^) are significantly smaller than the magnitude of the material intensity effect (on the order of ∼10 Mt year^–2^), indicating their lesser overall importance in changing the demand for concrete. The trend in the overall dwelling type effect is similar to the subeffect for detached houses in England, highlighting the importance of detached housing on demand for concrete: the value of the dwelling type subeffect for detached houses is positive from 1951 to 1991 and negative from 1992 to 2014. These periods are the same as those in which the proportions of detached new dwellings relative to the total number of new dwellings were increasing (1951–1991) and decreasing (1992–2014), respectively (Figure 5b). Since our results here show that reducing the proportion of detached dwellings in new construction corresponds to a reduction in the dwelling type effect, planners and policy makers can reduce the demand for concrete and associated environmental impacts by discouraging the construction of detached dwellings relative to other dwelling types.

Historically, values of the dwelling type subeffect for semidetached houses were negative before 2001, corresponding to a decreasing proportion of new semidetached type dwelling construction from 1951 to 2001 (Figure 5b). The results also show that the dwelling type subeffect for purpose-built flats is relatively small but shows an increasing trend from 1951 to 2014 (Figure 5a), which can be explained by the increasing proportion of purpose-built flats constructed during this period. These changes reflect the housing market and urban living conditions in England, which have generally trended toward smaller dwelling types and thus more compact urban environments, especially since the early 1990s when planning and design of dwellings better supported the construction of high-rise apartment blocks.^83^ Overall, there are a mixture of positive and negative contributions to the dwelling type effect, with its subeffect for detached housing dominating the overall trend.

### 3.4 Dwelling Intensity Effect

The dwelling intensity effect (*B*_DI_^*T*+1,*T*^) indicates the change in demand for concrete induced by the change in the number of new dwellings constructed per unit of GVA_C_. Dwelling intensity (DI) indicates how the number of new dwellings changes with the GVAc. Therefore, dwelling intensity shows how well established the dwelling stock (and thus demand for dwellings and building materials) is in a particular region and time period with reference to its level of construction sector activity. We explain dwelling intensity in general terms as follows: At low construction sector activity (low GVA_C_, corresponding to less developed economies), the dwelling stock is not saturated, and thus relatively more construction is for dwellings (to fulfill the basic service of shelter) that are demanded as the economy develops. However, at high construction sector activity (high GVA_C_, corresponding to more highly developed economies), the dwelling stock approaches saturation, and thus more demand for new dwellings exists to replace existing dwellings that have reached end-of-life rather than increase the total number of dwellings in the stock, and relatively more construction is for nonresidential buildings and infrastructure. We discuss the dwelling intensity effect and dwelling intensity further in Supporting Information S1 (Appendix S7).

**Figure 6:**
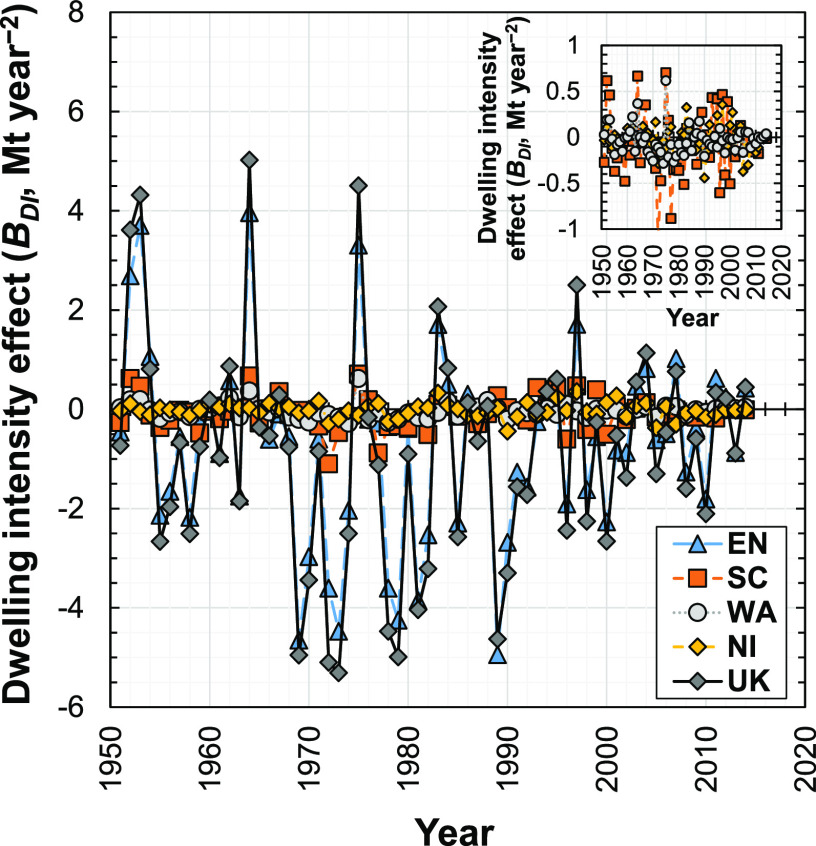
Influence of the dwelling intensity effect on the change in demand for concrete in new dwellings in the U.K. and its four subregions (EN, England; SC, Scotland; WA, Wales; NI, Northern Ireland; U.K., United Kingdom) from 1951 to 2014.

The dwelling intensity effect for the U.K. fluctuates between about ±5.0 Mt year^–2^ from 1951 to 2014 (Figure 6). The years with the largest dwelling intensity effect values and relatively larger numbers of new dwellings constructed (relative to the trend) are similar (Figure S2d, Supporting Information S1); the peaks in Figure 6 correspond to periods of high dwelling construction activity. For example, the sharp increase in construction of dwellings in post-World War II may explain the peaks in the early 1950s.^84,85^ The magnitudes of these peaks tend to decrease slightly over time, which are generally smaller after year 2000 than those in the 1900s.

Similar to the other effects, England dominates the dwelling intensity effect relative to the three other U.K. subregions (Figure 6). The dwelling intensity effects for the other subregions are similar in magnitude. The trends of the dwelling intensity subeffects all fluctuate in nature. A similar fluctuating trend in technology (material stock intensity per GDP) for Japan’s prefectures^55^ has been observed, indicating that it is not U.K.-specific.

### 3.5 Economic Output Effect

**Figure 7:**
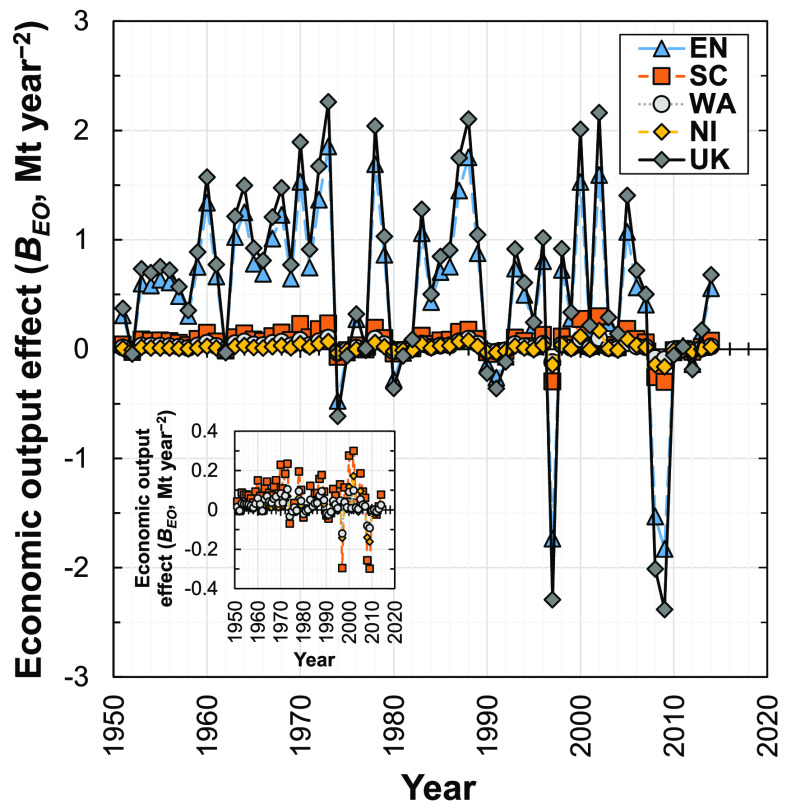
Influence of the economic output effect on the change in demand for concrete in new dwellings in the U.K. and its four subregions (EN, England; SC, Scotland; WA, Wales; NI, Northern Ireland; U.K., United Kingdom) from 1951 to 2014.

The economic output effect (*B*_EO_^*T*+1,*T*^) in the U.K. and its subregions fluctuated during 1951–2014 (Figure 7) but cumulatively increased the demand for concrete over this time period. Notable exceptions to this trend are observed in 1997 and 2008–2009, with a decrease in demand for concrete in new dwellings of 2.4 Mt year^–2^ determined in 2009 (Figure 7). Therefore, growth of the U.K. construction sector (Supporting Information S1, Appendix S3) has generally induced an increase in demand for concrete since 1950.

England dominates the economic output effect relative to the other three subregions (Figure 7), although all subregions show similar fluctuating trends. Various researchers have identified GDP per capita to be an important driver for material use.^40,55^ However, the economic output effect has the third largest effect on concrete demand here. This indicates that the rate of change of GVA_C_^86^ per capita had a lesser influence on the change in demand for concrete (and thus likely demand for building materials) than the material intensity and dwelling intensity effects. Additionally, in comparison to an *IPAT* type decomposition, our analysis decomposes the technology (*T*) term into four components (material intensity, MI; floor area shape, FAS; dwelling type, DT; dwelling intensity, DI): the effects induced by these drivers partially cancel each other out (e.g., the material intensity effect is positive, whereas the average dwelling intensity effect is negative).

### 3.6 Population Effect

**Figure 8:**
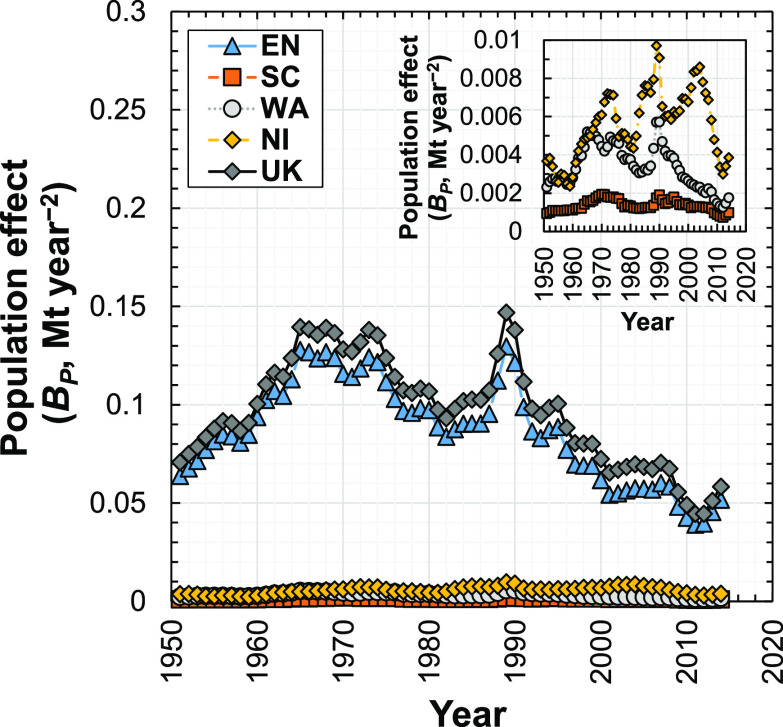
Influence of the population effect on the change in demand for concrete in new dwellings in the U.K. and its four subregions (EN, England; SC, Scotland; WA, Wales; NI, Northern Ireland; U.K., United Kingdom) from 1951 to 2014.

The population effect (*B*_*P*_^*T*+1,*T*^) is positive from 1950 to 2014 (Figure 8), which shows that population growth increased the demand for concrete in new dwellings in the U.K. and its subregions during this period. However, the population effect has a maximum value of 0.15 Mt year^–2^ in 1989. This is 2 orders of magnitude lower than the most dominant effects (material intensity, dwelling intensity, economic output), meaning that for economies like the U.K. where population growth is small (≤ ∼1% p.a. between 1951 and 2014), population change is a relatively insignificant driver of demand for building materials. This finding was also observed for Japan.^55^ Therefore, our results contrast the use of population as a main driver for modeling demand for building materials in developed (e.g., the U.K.^87^) and developing economies (e.g., China,^88^ Brazil^26^). Another possible reason for the relatively insignificant population effect on demand of concrete in the U.K. is that this research focuses on national level rather than city level. The rapid growth of population in megacities has been considered as a major driver for the demand for materials, so different results may be expected on the city scale.^35^

The population effect for England is generally an order of magnitude larger than the corresponding values for the other U.K. subregions (Figure 8), which is consistent with the much larger population of England (∼54 million persons in 2014) compared to those in other U.K. subregions (Scotland, ∼5 million persons in 2014; Wales, 3 million persons in 2014; Northern Ireland, ∼2 million persons in 2014; Supporting Information S1, Appendix S3).

### 3.7 Comparison of Individual Effects

**Figure 9:**
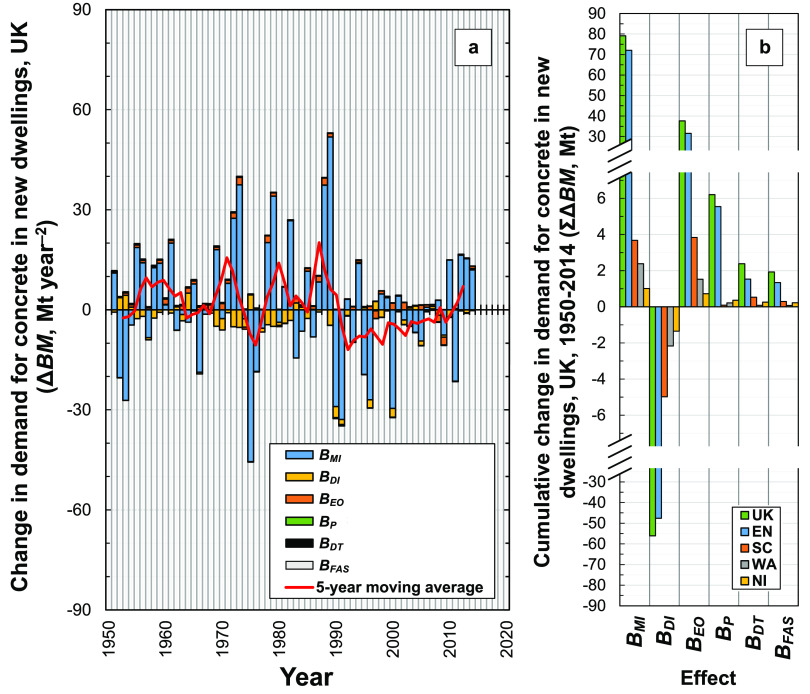
Overall contributions of the six different effects on the (a) year-on-year change in demand (U.K.) and (b) cumulative change in demand (U.K. and its four subregions: EN, England; SC, Scotland; WA, Wales; NI, Northern Ireland), for concrete in new dwellings from 1951 to 2014. The following abbreviations are used: *B*_MI_, material intensity effect; *B*_FAS_, floor area shape effect; *B*_DT_, dwelling type effect; *B*_DI_, dwelling intensity effect; *B*_EO_, economic output effect; *B*_*P*_, population effect. The 5-year moving average is for the total of all six effects.

We compare the cumulative contributions of these six effects on changes in demand for concrete in new dwellings in the U.K. during 1951–2014 in Figure 9. During this period in the U.K., changes in material intensity were responsible for +79 Mt concrete demand (+1.2 Mt year^–2^ on average, Figure 9b). It is by far the most important effect, with its values in any given year generally much larger in magnitude than the other effects. The second most important effect is the dwelling intensity effect, which had a cumulative negative value of −56 Mt during 1951–2014 (−0.88 Mt year^–2^ on average), and third the economic output effect, which had a cumulative positive value of +38 Mt during 1951–2014 (+0.59 Mt year^–2^ on average). The cumulative values of these three effects are thus all within the same order of magnitude. Therefore, although the material intensity effect results in large year-to-year increases and decreases in concrete demand (Figure 9a), over the time period of 1951–2014 its cumulative effect to increase demand for concrete is significantly offset by the dwelling intensity effect in the U.K. On this same cumulative basis, the other effects (floor area shape effect, dwelling type effect, and population effect) have at least an order of magnitude smaller values. Therefore, it is important to include the former three effects but especially the material intensity effect in analyses of demand for building materials in the U.K., e.g., in dMFA and IPAT analysis, and likely also in studies of other regions. The three latter effects may be situationally important: population changes are likely to be important in economies with relatively high population growth; and dwelling size (floor area) and style (dwelling type) are key drivers of energy demand and related emissions of dwellings in the U.K.,^89,90^ Australia,^91^ and China.^92^

The material intensity effect in England is the most important of all effects in inducing demand for concrete, which alone induces about +72 Mt of concrete demand during 1951–2014 (+1.1 Mt year^–2^ on average, Figure 9b). In dMFA studies, material intensity has also been used to assess building material stocks, such as concrete, cement, and timber.^33,85,93^ Cumulatively, the dwelling intensity effect is the most negative of all effects and thus plays an important role in reducing demand for concrete. For example, in England, demand for concrete was reduced by ∼48 Mt due to the dwelling intensity effect from 1951 to 2014 (−0.74 Mt year^–2^ on average).

Year-on-year changes in demand for concrete in new dwellings in the U.K. fluctuate from 1950 to 2014 (Figure 9a, bars), although on average the values tend to be more positive before 1990 and less positive or negative thereafter (Figure 9a, line). This fluctuating trend explains our result that the yearly demand for concrete, both in new dwellings (15–40 Mt year^–1^) and overall (50–130 Mt year^–1^), has remained positive and relatively steady (around the respective average values of ∼25 Mt year^–1^ and ∼85 Mt year^–1^) during 1950–2014 (Figure S3, Appendix S4, Supporting Information S1). Since over this time period the U.K. population grew slowly, from 49.7 million persons in 1950 to 64.6 million persons in 2014, yearly per capita demand for concrete in dwellings and overall in the U.K. have remained at ∼0.5 t year^–1^ and ∼1.5 t year^–1^, respectively. The year-on-year changes in demand for concrete in new dwellings that we calculate (Figure 9a, lines) are partly consistent with both “linear growth” (type I) and “decelerating” (type IV) stock accumulation patterns defined by Fishman et al.^94^ Therefore, although Fishman et al. assigned the U.K. to the type IV pattern, our results are generally consistent with their analysis. We attribute the difference in interpretation to our analysis including data up to year 2014, because the year-on-year changes in demand for concrete only become significantly positive from 2010, which their study did not include (it analyzed data for the time period 1970–2010). Analysis of more recent data would thus be beneficial to confirm the recent accumulation pattern of the building material stock in the U.K.

## 4 Perspectives

The LMDI approach focuses on exogenous (characteristics of society, e.g., GVA_C_ and population) and endogenous (characteristics of dwellings, e.g., types and floor area of dwellings) drivers to analyze the changes that they induce over time. These drivers have been partly analyzed before in the context of building materials: for material intensity, industrial structure (the total inputs into a sector for a unit of final product from a sector), and population in Australia;^95^ for steel–concrete and masonry–concrete structures, the amounts of dwellings, and use of materials per unit building floor area in Beijing;^96^ for GDP and population in Japan;^55^ and for population, fixed investment, and cement consumption intensity (cement consumption per GDP) in China.^40^ However, our paper provides a comprehensive analysis of how these drivers affect the demand for concrete, in space (U.K.) and time (1951 to 2014).

Our subregional analysis shows that their effects in England dominate the U.K. subregions. Therefore, we demonstrate the strong influence of spatial distribution on the demand for concrete, and likely also other building materials. This result indicates that further spatial disaggregation (e.g., to county/city or urban/rural levels) would be beneficial in understanding and analyzing building material demand and use and highlights the important role that municipalities (e.g., cities) play in transitioning toward sustainable development.

Regional differences in the drivers of building material use have been discussed for Japanese prefectures^55^ and Chinese cement consumption,^40^ but not using the additive LMDI method. Application of the LMDI method to these and other countries, such as the U.S. and Australia, may thus illuminate the key effects on building material demand and stocks in a multinational context. This insight can then be used to define and quantify measures that may lead to global environmental benefits, including construction with preferential material substitutions, different building types, and changes to economic output. Here, we show that such measures act to accelerate or decelerate growth of the in-use stock;^94^ e.g., material substitution changes the demand for a building material, which in turn changes the level of the in-use stock of that building material.

Our results clearly show that the material intensity effect induces the largest demand for concrete in the U.K., and thus it will likely also do so for similar economies. Therefore, material intensity is a key parameter to include in modeling of stock-flow dynamics of building materials. For the exogenous drivers, the economic output effect has the second important positive effect on demand for concrete. Therefore, economic output per capita is another important driver to include in modeling stock-flow dynamics of building materials. Overall, our LMDI method provides a new perspective in analyzing the stock-flow dynamics of materials used in the built environment, as an additional means to understanding relationships between socio-economic drivers, material consumption, and ultimately environmental impacts.

## Supporting Information Available

(S1) Appendix S1, calculation details from step one to step four for Figure 1 in the main text; Appendix S2, data gaps in the LMDI decomposition analysis; Appendix S3, trends in socio-economic drivers of concrete use; Appendix S4, sensitivity analysis for concrete intensity; Appendix S5, floor area shape subeffects; Appendix S6, correlation analysis between the dwelling intensity effect and the economic output effect; and Appendix S7, further discussion of dwelling intensity and the dwelling intensity effect (PDF)
(S2) Collection of data collected, generated, and used in this study (XLSX)
