# Supplementary material for: Log Mean Divisia Index Decomposition Analysis of the Demand for Building Materials: Application to Concrete, Dwellings, and the U.K
Source: Environ Sci Technol. 2021 Feb 20;55(5):2767–78. doi: 10.1021/acs.est.0c02387 (PMC8023668; doi:10.1021/acs.est.0c02387)
Supplement: Supplementary file 1 — es0c02387_si_001.pdf [file es0c02387_si_001.pdf]

Supporting Information for:

Log mean divisia index decomposition analysis of the demand for building materials: application to concrete, dwellings, and the UK

He He [1,2,a], Rupert J. Myers [1,2,b,*]

[1] Current address: Department of Civil and Environmental Engineering, Imperial College London, Skempton Building, London, SW7 2AZ, United Kingdom

[2] School of Engineering, The University of Edinburgh, King's Buildings, Sanderson Building, Edinburgh, EH9 3FB, United Kingdom

[*] Corresponding author. Email: [a] hhe1@ic.ac.uk; [b] r.myers@imperial.ac.uk.

Table of contents

S1. Calculation details from steps one to four in Figure 1 in the main text............................S3

S2. Data gaps in the log mean divisia index decomposition analysis....................................S5

S2.1. Data gaps in the fraction ($DT_j^{t,k}$) and floor area ($FAS_j^{t,k}$) of j type dwellings S5

S2.2. Data gaps in the gross value added of the construction sector...................................S6

S3. Trends in socio-economic drivers of concrete use S7

S4. Sensitivity analysis for concrete intensity S9

S4.1. Method and data sources...S10

S4.2. Results S12

S5. Floor area shape sub-effects S14

S6. Correlation analysis between the dwelling intensity effect and the economic output effect S16

S7. Further discussion of dwelling intensity and the dwelling intensity effect S17

S8. References in this supporting information document S22

S1. Calculation details from steps one to four in Figure 1 in the main text

This section elaborates the calculation details involved in steps one to four shown in Figure 1 in the main text. In step one, dwelling types were classified as bungalow house, detached house, semi-detached house, mid terrace house, end terrace house, purpose-built flat, and converted flat, consistent with the classifications of the English Housing Survey and Scottish House Condition Survey.[1,2] The amounts of j type dwellings constructed in each region k and at time t analysed ($DW_j^{t,k}$, dwellings year^{-1}) were determined by multiplying the total amounts of dwellings constructed at a given time t ($DW^{t,k}$, dwellings year^{-1}) by the fractions of j type dwellings constructed ($DT_j^{t,k}$, dimensionless) in that same and region and time (i.e., year) (eq.(S1)):

$$DW_j^{t,k} = DW^{t,k} \times DT_j^{t,k} \tag{S1}$$

In step two, the total floor area ($FA_j^{t,k}$, m^2 year^{-1}) of j type dwellings constructed in each region k and at time t were calculated by multiplying the floor area of each dwelling type ($FAS_j^{t,k}$, m^2 dwellings^{-1}) by $DW_j^{t,k}$, which has the same definition as above (eq.(S2)):

$$FA_j^{t,k} = FAS_j^{t,k} \times DW_j^{t,k} \tag{S2}$$

In step three, key building materials used were identified to be cement, concrete, steel, wood, sand, gravel, glass, brick, and ceramics.[3] The amounts and prices of these building materials are related to economic changes (e.g., the development of the gross value added of the construction sector (GVA_C) per capita).[4] We calculated the amount of building material i used in j type new dwellings in region k and at time t ($BM_{i,j}^{t,k}$, Mt year^{-1}) by multiplying the

concrete intensity of building material *i* per floor area ($MI_{i,j}{}^{t,k}$, Mt m^{-2}) by $FA_j{}^{t,k}$, in new dwellings which has the same definition as in eq.(S2) above:

$$BM_{i,j}^{t,k} = MI_{i,j}^{t,k} \times FA_j^{t,k} \quad \text{(S3)}$$

In step four, we included GVA_C and population as socio-economic drivers to describe the demand for building materials. The sum of GVA and taxes on products is equivalent to gross domestic product (GDP).

S2. Data gaps in the log mean divisia index decomposition analysis

S2.1. Data gaps in the fraction ($DT_j^{t,k}$) and floor area ($FAS_j^{t,k}$) of *j* type dwellings

The fraction ($DT_j^{t,k}$) and floor area ($FAS_j^{t,k}$) of *j* type dwellings constructed in a given year *t* in England and Scotland were taken from the English Housing Survey and Scottish House Condition Survey, respectively.[1,2] Comparable datasets are unavailable for Wales and Northern Ireland. Therefore, we applied the following two assumptions to construct datasets for those countries: (1) for Wales, values of $DT_j^{t,k}$ for England and $FAS_j^{t,k}$ for Scotland were used; and (2) for Northern Ireland, values of $DT_j^{t,k}$ for Scotland and $FAS_j^{t,k}$ for England were used. These assumptions do not significantly affect our results and analysis since England and Scotland dominate the trends in construction of new dwellings in the UK. The average floor area per dwelling type from 1950 to 2014 in the UK is shown in Figure S1.

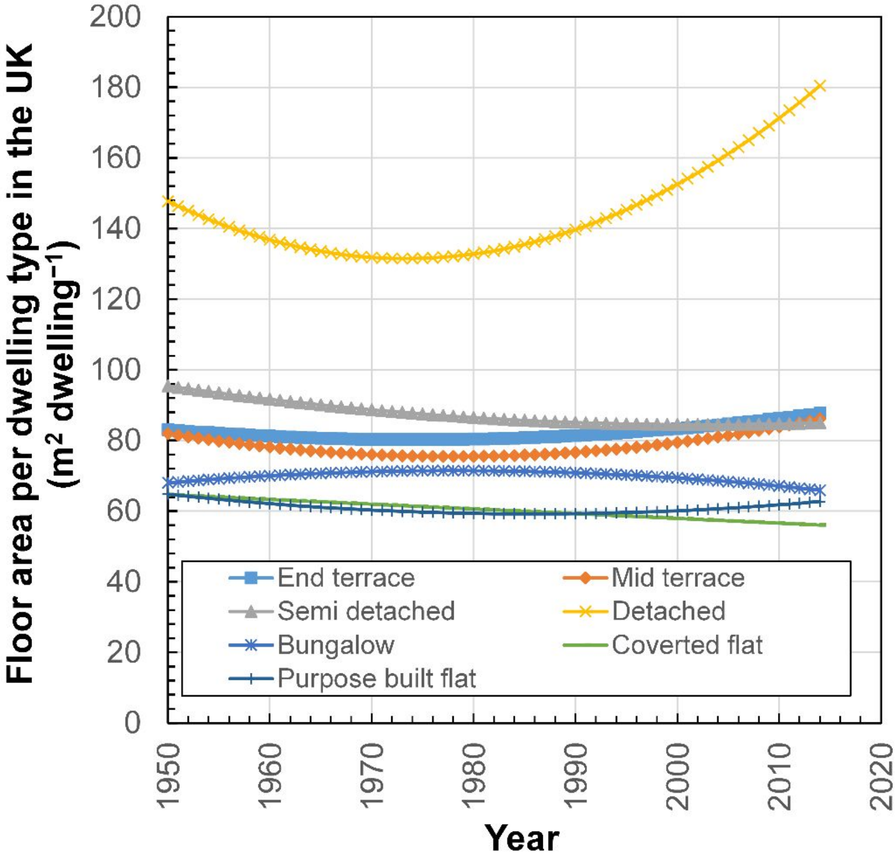

Figure S1. Average floor area per dwelling and type from 1950 to 2014 in the UK.

S2.2. Data gaps in the gross value added of the construction sector

Data gaps for the GVA_C in the four UK sub-regions (England, Scotland, Wales, Northern Ireland) were estimated using regression models. The estimation process includes three steps:

1. We estimated the GVA_C in the UK through a linear regression model based on data from the Office for National Statistics.[5,6]
2. We calculated the proportion of GVA in the four UK sub-regions.[7]
3. We multiplied the GVA_C in the UK with the proportions of GVA in each UK sub-region to obtain the GVA_C in the four UK sub-regions.

The estimated results for GVA_C in the four UK sub-regions are shown in Data Sheet 9 in Supplementary Information S2.

S3. Trends in socio-economic drivers of concrete use

Changes to the UK population and GVA_C affect the rate of construction of new dwellings at this national level.[8] The growth in UK population accelerated from 1951 to 1963, peaking at ~+1% in 1962, decelerated until it stabilised at around 1980, and then accelerated again from 1983 to 2014 (Figure S2a, inset). The growth in the deflated GVA_C and the number of dwellings in the UK fluctuated from 1951 to 2014 (Figure S2a).

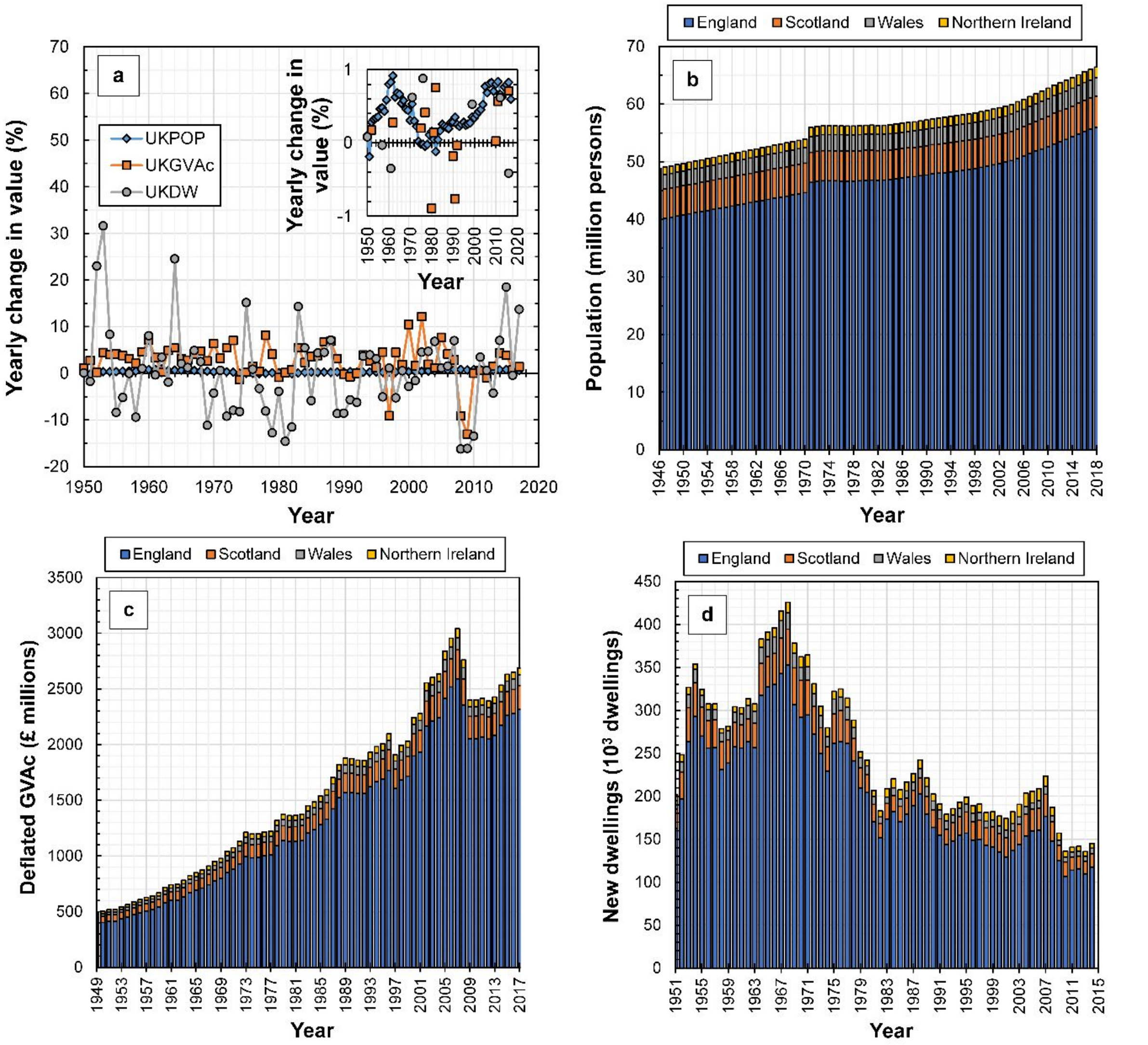

Figure S2. Socio-economic drivers of new dwelling construction in (a) the UK and (b-d) its four sub-regions (England, Scotland, Wales, Northern Ireland): (a) change in population, GVA_C (deflated), and number of new dwellings constructed; (b) population; (c) GVA_C (deflated); (d) number of new dwellings constructed. UKPOP is the UK population;

UKGVA_C is the GVA_C of the UK construction sector; UKDW is the number of new dwellings constructed in the UK (in a given year).

The population in England grew steadily between 1951 and 2014 while other three sub-regions (Scotland, Wales, and Northern Ireland) remained stable over this period (Figure S2b). The population in England is the largest of all UK sub-regions (Figure S2b). The GVA_C of England increased from ~£0.38 billion in 1948 to ~£2.6 billion in 2007, while the values for the other three sub-regions showed much smaller increases in magnitude during this period (Figure S2c). The GVA_C in all sub-regions peaked in 2007; the largest reduction in GVA_C occurred in the following period, of £541 million between 2007 and 2009, in England (Figure S2c). The number of dwellings constructed in England fluctuated significantly from year-to-year between 1951 and 2014, peaking at 352,540 new units constructed in 1968 (Figure S2d). Similar fluctuations in the number of dwellings constructed in the other three sub-regions occurred during the period. These data show that the contributions of England to the UK values and trends are much greater than contributions from the other three UK sub-regions.

S4. Sensitivity analysis for concrete intensity

Sensitivity analysis is a technique used to assess how the uncertainty in the output of a model can be apportioned to different sources of uncertainty in the model input.[9] The Monte Carlo method, one of the major methods for sensitivity analysis, has been used to assess uncertainty relating to the level of aggregation,[10] total CO_2 emission intensities,[11] impacts of models on eco-efficiency assessment,[12] technical waste and primary input coefficients,[13] and parameter values in hybrid life cycle assessment.[14]

Here, we apply the following method to calculate the (average) inflow concrete intensity in the UK (Figure S3; here inflow refers to the flow into use, which is equivalent to demand). We do this recognising that the inflow concrete divided by inflow (new) floor area is equivalent to the inflow concrete intensity. To be more precise, we obtained the inflows of concrete in the UK from 1800 to 2017.[15] Concrete has three main uses: residential buildings (dwellings), non-residential buildings, and civil engineering.[16] We consider the data for new residential buildings (dwellings) in the UK here. We thus calculate the inflows of concrete in the UK from 1951 to 2014 for residential buildings (dwellings) by multiplying the inflows of concrete in the UK with the proportion of cement used in residential buildings (dwellings). The total inflow of floor area in new dwellings in the UK is the sum of the inflow of floor area in seven types of new dwellings. We summarise these data in Figure S3.

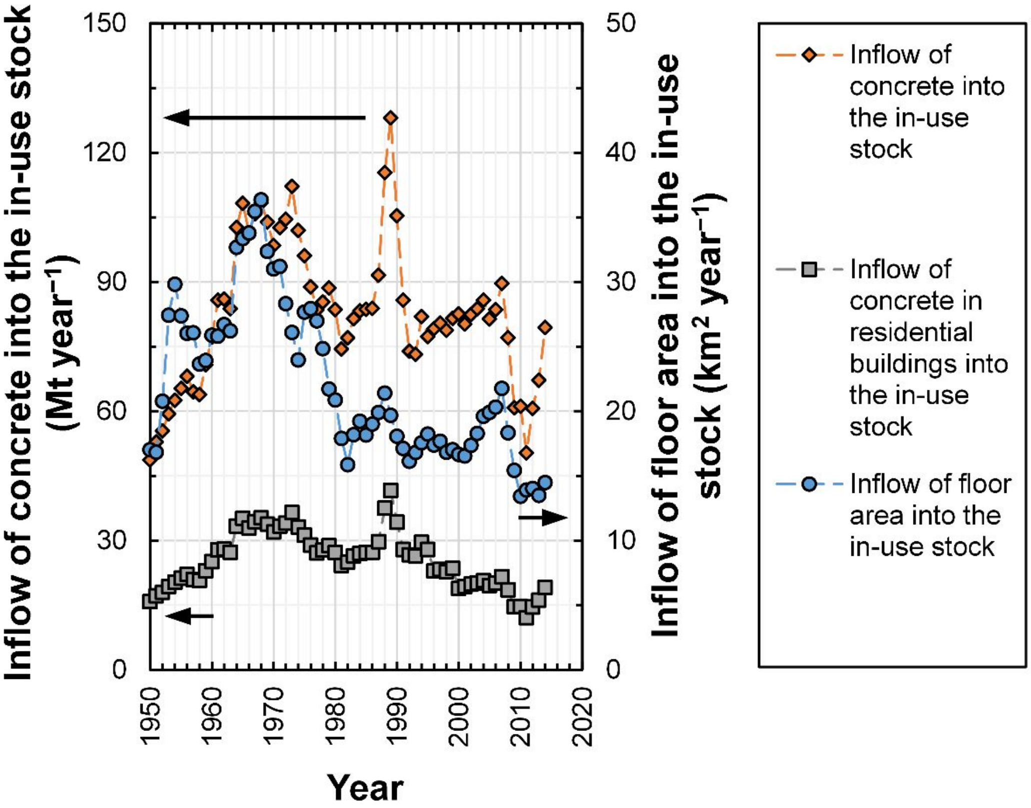

Figure S3. Inflows of concrete and floor area into the in-use stock of dwellings in the UK from 1950 to 2014. The arrows are guides for the eye.

Due to the limited data for concrete intensity in the UK, we conducted a sensitivity analysis to determine how significantly concrete intensity impacts the changes in demand for concrete that we calculate using the logarithmic mean divisa index (LMDI) method under a given set of assumptions.

S4.1. Method and data sources

We conducted our sensitivity analysis along the following three steps. The first step was to generate 10 random samples of concrete intensities based on three samples of concrete intensity values in Sweden, Norway, and the UK in each year. Concrete intensities in Sweden,[17] Norway,[18] the Netherlands,[19] the UK (average, 'estimated') and UK (actual values for specific buildings, 'observed') are shown in Figure S4.

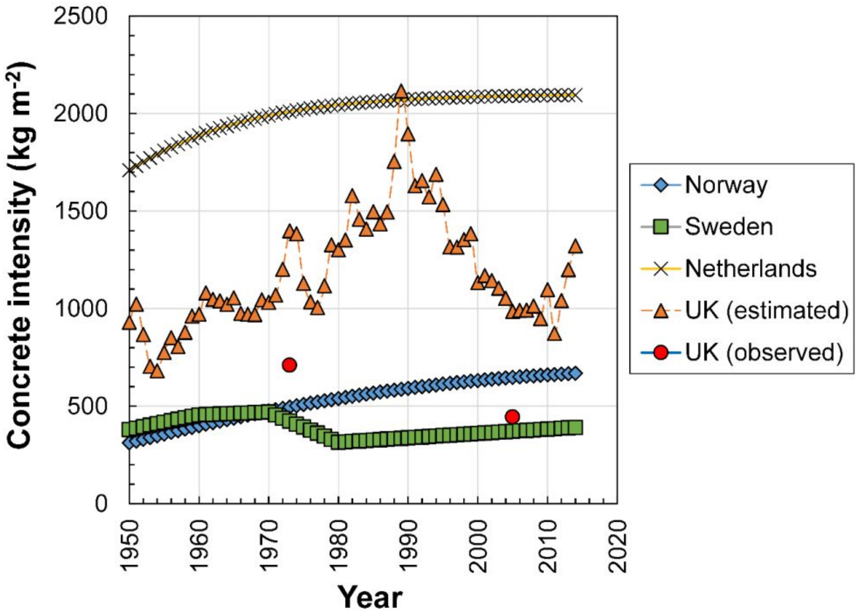

Figure S4. Concrete intensities in Norway, Sweden, the Netherlands, the UK (average, 'estimated'), and UK (actual values for specific buildings, 'observed') in buildings from 1951 to 2014.

We generate ten random samples of concrete intensity values based on the samples in these three countries (Norway, Sweden, the Netherlands, and the UK). The first step is to apply the method[20] to calculate adjusted means and standard deviations using a truncated normal distribution (allowing only positive values). We do this by using the iterative eqs.(S4-S7), which are defined in terms of the means and standard deviations of the concrete intensities in Norway, Sweden, and the UK. The truncated normal distribution is used as concrete intensity values cannot be negative; we allowed all positive values.

$$\alpha_n = (B - \mu_n)/\sigma_n \quad \text{(S4)}$$

$$\beta_n = (C - \mu_n)/\sigma_n \quad \text{(S5)}$$

$$\mu_{n+1} = \bar{X} + \sigma_n(\phi(\beta_n) - \phi(\alpha_n))/(\Phi(\beta_n) - \Phi(\alpha_n)) \quad \text{(S6)}$$

$$\sigma_{n+1}^2 = S^2 + (\bar{X} - \mu_{n+1}) + \sigma_n^2(\beta_n\phi(\beta_n) - \alpha_n\phi(\alpha_n))/(\Phi(\beta_n) - \Phi(\alpha_n)) \quad \text{(S7)}$$

In eqs.(S4-S7), subscript n denotes the nth approximation to the maximum likelihood estimate, B and C are the maximum and minimum numbers of the collected sample, ϕ and Φ refer to the ordinate and cumulative area of the unit normal curve, $\bar{X}$ is the average of the collected data, and S is the standard deviation of the collected data. We found that after five iterations, stable estimates of the mean and standard deviation, μ_n and σ_n, were reached.

The second step is to apply the stable μ_n and σ_n values to obtain ten random samples of the concrete intensity, and then to input the samples into the LMDI method to determine the corresponding changes in concrete demand. We chose seven years to apply this random sampling procedure (1954, 1964, 1974, 1984, 1994, 2004, 2014) to in our sensitivity analysis. We thus input these seven concrete intensity values into our LMDI method to obtain the material intensity (kg material m^{-2} floor space) effects. The last step is to calculate the coefficient of variation of the change in demand for concrete caused by the material intensity effects to assess the uncertainty in the input data.

S4.2. Results

In this section, we display the results based on the calculation of three steps mentioned in Section S4.1. Figure S5 displays box-and-whisker plots of the estimated concrete intensity values for the ten samples (labelled 1-10).

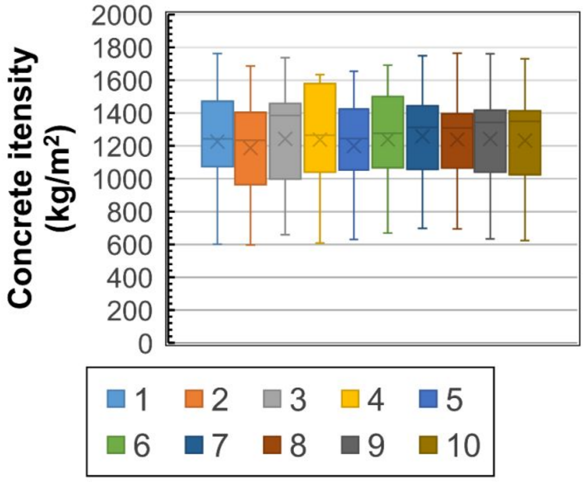

Figure S5. Box-and-whisker plots of the concrete intensities in buildings estimated in ten random samples (1-10).

We then inputted the replaced samples into the LMDI method at the seven years (1954, 1964, 1974, 1984, 1994, 2004, 2014) to obtain the corresponding changes in demand for concrete in the UK. We calculated coefficients of variation for these corresponding changes in demand for concrete. Here, a coefficients of variation represents a ratio of the standard deviation of the estimated change in demand for concrete to the mean of the estimated change in demand for concrete. Coefficients of variation were determined to generally be between the range [–9.3%, +8.8%] (Table S2), which is comparable to the values in similar research (e.g. Glöser et al. (2013)),[21] indicating an acceptable level of uncertainty in concrete intensity values for the UK that we use here.

Table S2. Coefficients of variation for the changes in demand for concrete (%) in different years, new dwellings, and the UK.

Year	Coefficients of variation of the change of concrete demand (%)
1954	-5.3
1964	6.6
1974	-9.3
1984	-7.8
1994	6.5
2004	-7.4
2014	8.8

S5. Floor area shape sub-effects

We investigated the floor area shape effect by disaggregating it into sub-effects for different dwelling types, since distinct dwelling types have different characteristic floor areas and thus demand for concrete. Therefore, we disaggregated the floor area shape effect in England into seven sub-effects, representing floor area shape sub-effects for bungalow houses, detached houses, mid-terrace houses, purpose-built flats, end-terrace houses, semi-detached houses, and converted flats (Figure S6).

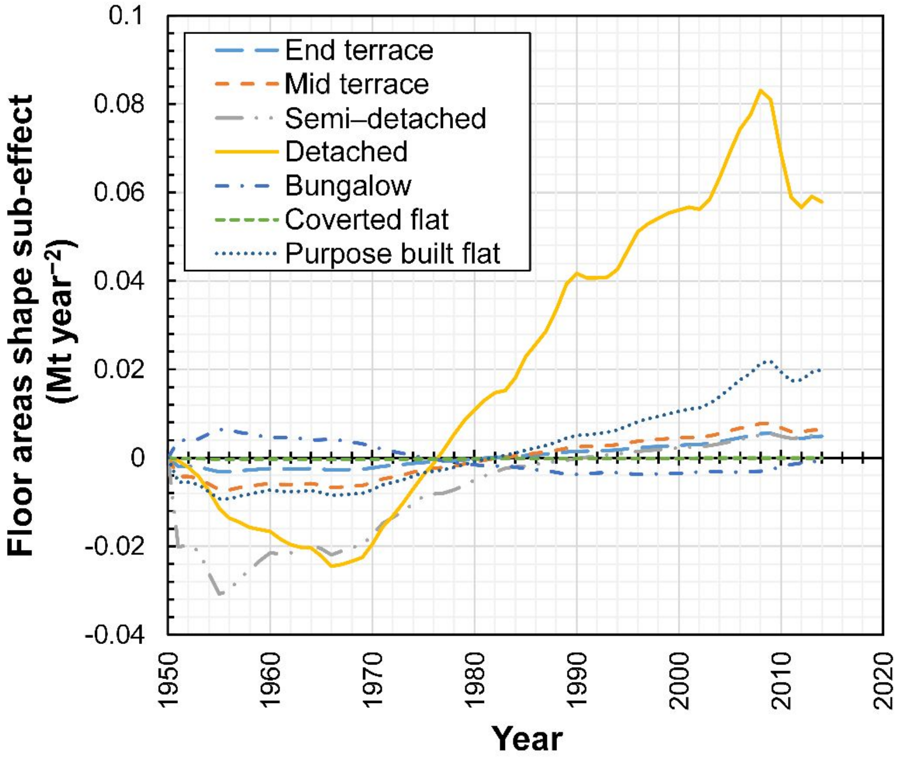

Figure S6. Influence of the floor area shape sub-effects on the demand for concrete in the seven types of new dwellings in England during 1951–2014.

The magnitudes of sub-effects for detached and semi-detached dwelling types were generally larger than the other dwelling types during before 1980, leading to an overall negative floor area shape effect in this time period (Figure 3 in the main text). From 1980 onwards, the (positive) floor area shape sub-effect of detached dwellings dominated the sub-effects for the

other dwelling types, although purpose-built flats also showed a relatively high floor area shape sub-effect during this period. The floor area shape sub-effect for bungalows shows an opposite trend to that for the other dwelling types, which is positive before 1976 and negative after 1976. Overall, our results show that the floor area shape sub-effect for detached houses provides the major contribution to changing the demand for concrete relative to the other sub-effects, which is consistent with comparative studies of material use in UK buildings.[22] Therefore, measures that change the floor area of detached houses, such as the movement towards 'tiny houses', are most likely (relative to other dwelling types) to significantly affect demand for concrete.

S6. Correlation analysis between the dwelling intensity effect and the economic output effect

We perform a regression analysis to explore correlations between all effects in our study (presented in full in Data Sheet 28, Supplementary Information S2). Here, as an example of our complete analysis, we show the results from our linear regression analysis[23] for X variable (dwelling intensity effect) and Y variable (economic output effect) to check if there is any correlation between these variables. The result is shown in Table S3 with 'X Variable' as the dwelling intensity effect and 'Y Variable' as the economic output effect. The R-squared is 0.06013, which indicates that ~6% of economic output effect can be explained by the dwelling intensity effect. The p-value for 'X Variable' (dwelling intensity effect) is 0.05082, which is larger than 0.05. This indicates that there is not a statistically significant relationship between 'X Variable' (dwelling intensity effect) and the 'Y Variable' (economic output effect).

Table S3. Results from the correlation analysis between the dwelling intensity effect and the economic output effect. 'X Variable' is the dwelling intensity effect and 'Y Variable' is the economic output effect.

Multiple R	0.24522					
R Square	0.06013					
Adjusted R Square	0.04497					
Standard Error	0.46588					
Observations	64					
	Coefficients	Standard Error	t Stat	P-value	Lower 95%	Upper 95%
Intercept	0.1813	0.0637	2.845	0.006	0.0539	0.3086
X Variable	-0.1295	0.0650	-1.991	0.0508	-0.259	0.0004

S7. Further discussion of dwelling intensity and the dwelling intensity effect

The purpose of a decomposition analysis is to disaggregate an important indicator (e.g. the amount of energy or carbon emissions) into other important yet easier to understand indicators (drivers). The essence of the LMDI method is to understand resource use from three perspectives: K, investment; L, labor; and T, technology.[24] For example, the material intensity effect and floor area shape effect represent the effects of technology on resource use. The dwelling intensity effect and economic output effect represent the effects of investment on resource use. The population effect represents the effect of labor. Many LMDI studies cover these three perspectives, since they tend to all be important.[25,26]

In our paper, $B_{DI}^{T+1,T}$ (tonnes year^{-2}) is the dwelling intensity effect, which relates changes in the number of new dwellings per GVA_C to changes in the demand for building material *i*. The mathematical equation of the dwelling intensity effect is shown in Table 2 (main text). The core driver embedded in the dwelling intensity effect is the dwelling intensity (*DI*) indicator, which is defined as the number of new dwellings per GVA_C (Table 1, main text). It is analogous to energy intensity, which is a widely used indicator to reflect the energy efficiency of an economy.[27]

Dwelling intensity can be thought of as a relative measure of how much construction sector economic activity is translated into construction of new dwellings in an economy. It indicates how well established the dwelling stock (and thus inflows into the in-use stock) is in a specific region and time with reference to the level of economic activity in the region's construction sector. This is because for a relatively less developed economy, there is greater demand for new (additional) housing to upgrade its dwelling stock, whereas for a relatively highly

developed economy, there is rather more demand to replace existing dwelling stock that has reached end-of-life. As an economy develops its dwelling stock will usually eventually approach saturation, which explains this transition from a drive to increase the total dwelling stock towards a drive to replace end-of-life dwellings.

From the perspective of a nation's economy, a theoretical relationship exists between the GVAc and building permits, and thus new construction of buildings and dwellings.[28] From a practical perspective, the government forecasts the next year's GVAc. Therefore, once we know the dwelling intensity, we can estimate how many new dwellings should be constructed from a GVA_C forecast, which supports the government to issue building permits in the future. These examples demonstrate the practical relevance of the dwelling intensity indicator.

In this section we test the relationship between the number of new dwellings constructed and the GVAc in the UK (Figure S7). The exponential relationship (Figure S7, blue line) between the GVAc and the number of new dwellings has the highest R-squared (0.6349), which indicates that 63.49% of the number of new dwellings are explained by the GVAc using this relationship. As discussed above, an exponential relationship is justified conceptually through the concept of in-use stock saturation, which is a well-known phenomena.[29]

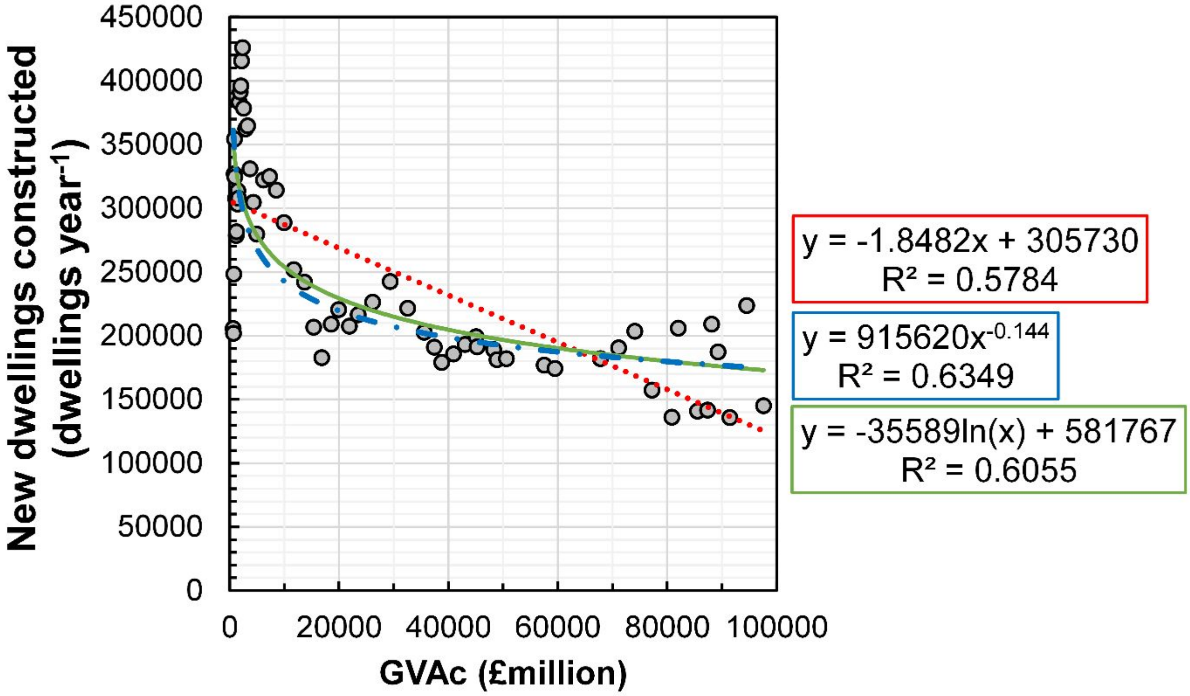

Figure S7. Relationship between the GVAc and the number of new dwellings constructed. The equations for the lines plotted are shown.

An increase in the GVAc results in a decrease in the number of new dwellings before the number of dwelling stock reaches saturation (Figure S7, GVA_C < ~£30,000 million). When the number of dwellings in the stock reaches saturation, the number of new dwellings constructed will remain stable with increasing GVA_C. Therefore, when the population of an economy reaches a stable level, the number of new dwellings will be equal to the demolished dwellings. The functions of dwellings are special relative to other goods, because in the absence of some particular cases (e.g., wars, natural disasters, poor quality construction), dwellings can have very long lifetimes in use. At population saturation, the number of new dwellings will decrease to reach 'equilibrium' with (equal to) the demolition rate (mass conservation; the change in the in-use stock is zero).

At the saturation level, the slopes of the non-linear trendlines in Figure S7 (i.e., *DI*) approach zero. This will result in the term $ln(\frac{DI^{T+1,k}}{DI^{T,k}})$ becoming negative in value. Therefore, the

dwelling intensity effect will become negative with the technology factor $L(BM_i^{t_T+1,k},BM_i^{t_T,k})$ keeping stable. This is why the cumulative contribution of the dwelling intensity effect is negative over the time period 1950-2014 (Figure 9b, main text).

In developing the dwelling intensity indicator, we considered the possibility of including house prices in our decomposition. In Figure S8 we show the relationship between the dwelling intensity effect and new house prices from 1952 to 2014 in the UK. There is no clear relationship between the dwelling intensity effect and the new house price. One reason why there is no relationship between the dwelling intensity effect and the house price is because the former relates to the economic activity of the construction sector whereas the latter relates to the economic activity of the real estate sector (which includes sales of both new and old dwellings). House prices depend greatly on immaterial factors like location, whereas construction activity represents manufacturing of materials into products like buildings and so relates intimately to material stocks and flows.

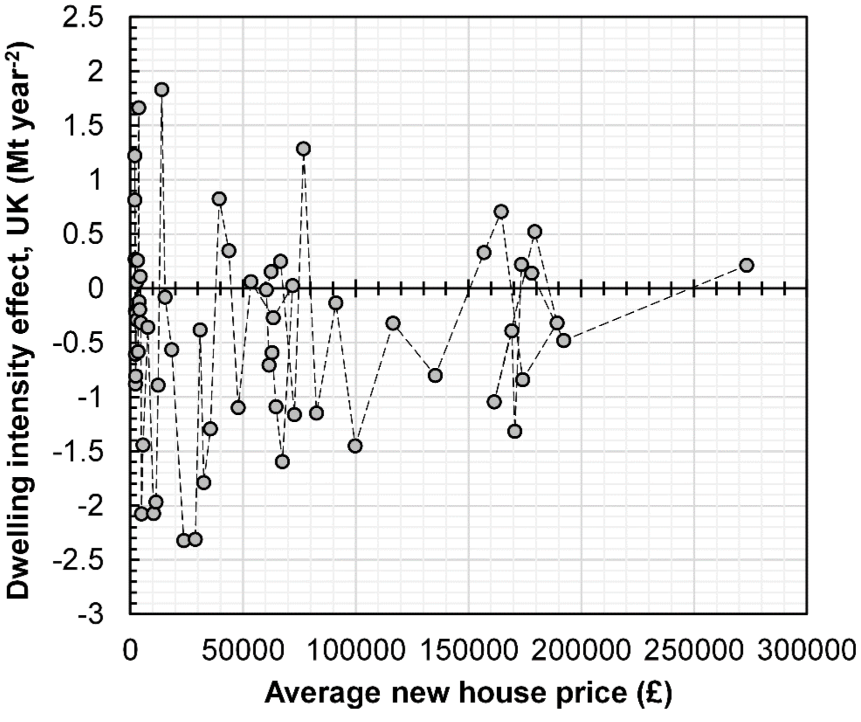

Figure S8. Relationship between the dwelling intensity effect and the average new house price in the UK from 1952 to 2014. Each datum represents a year; the lines show the time series and are intended as eye guides only.

S8. References in this supporting information document

(1) Ministry of Housing, Communities and Local Government. *English Housing Survey 2017 to 2018: Headline report*, 2019. <https://www.gov.uk/government/statistics/english-housing-survey-2017-to-2018-headline-report> (accessed: 10.10.19).

(2) Directorate for Housing and Social Justice Communities Analysis Division Scottish Government. *Scottish house condition survey: 2017 key findings*, 2018. <https://www.gov.scot/publications/scottish-house-condition-survey-2017-key-findings/> (accessed: 11 October 2019).

(3) Department for Business, Energy and Industrial Strategy. *Construction building materials: Bulletin, November 2019*, 2019. <https://assets.publishing.service.gov.uk/government/uploads/system/uploads/attachment_data/file/849710/19-cs12_-_Construction_Building_Materials_-_Bulletin_November_2019.pdf> (accessed: 21 November 2019).

(4) Davidson, E. Defining the trend: Cement consumption versus gross domestic product, *Global Cement Magazine*, **2014**, 8-14.

(5) Office for National Statistics. *Regional economic activity by gross value added (balanced), UK: 1998 to 2017*, 2018. <https://www.ons.gov.uk/economy/grossvalueaddedgva/bulletins/regionalgrossvalueaddedbalanceduk/1998to2017> (accessed: 21 November 2019).

(6) Office for National Statistics. *Gross value added (average) at basic prices: CP SA £m*, 2019. <https://www.ons.gov.uk/economy/grossvalueaddedgva/timeseries/abml/bb> (accessed: 22 November 2019).

(7) Office for National Statistics. *Regional economic activity by gross value added (balanced), UK: 1998 to 2017*, 2018 <https://www.ons.gov.uk/economy/grossvalueaddedgva/bulletins/regionalgrossvalueaddedbalanceduk/1998to2017> (accessed: 21 November 2019).

(8) Schmickler, A.; Park, K. S. UK social housing and housing market in England: A statistical review and trends. *LHI Journal*, **2014**, *5* (3), 193.

(9) Saltelli, A. (2002). Sensitivity analysis for importance assessment. *Risk Analysis*, *22* (3), 579-590.

(10) Bullard, C. W.; Sebald, A. V. Monte Carlo sensitivity analysis of input-output models. *The Review of Economics and Statistics*, **1988**, 708-712.

(11) Hondo, H.; Sakai, S.; Tanno, S. Sensitivity analysis of total CO_2 emission intensities estimated using an input–output table. *Applied Energy*, **2002**, *72* (3-4), 689-704.

(12) Egilmez, G.; Gumus, S.; Kucukvar, M.; Tatari, O. A fuzzy data envelopment analysis framework for dealing with uncertainty impacts of input–output life cycle assessment models on eco-efficiency assessment. *Journal of Cleaner Production*, **2016**, *129*, 622-636.

(13) Yazan, D. M.; Romano, V. A.; Albino, V. The design of industrial symbiosis: An input–output approach. *Journal of Cleaner Production*, **2016**, *129*, 537-547.

(14) Salemdeeb, R.; zu Ermgassen, E. K.; Kim, M. H.; Balmford, A.; Al-Tabbaa, A. Environmental and health impacts of using food waste as animal feed: A comparative analysis of food waste management options. *Journal of Cleaner Production*, **2017**, *140*, 871-880.

(15) Streeck, J.; Wiedenhofer, D.; Krausmann, F.; Haberl, H. Stock-flow relations in the socio-economic metabolism of the United Kingdom 1800–2017. *Resources, Conservation and Recycling*, **2020**, *161*, 104960.

(16) Cao, Z.; Shen, L.; Løvik, A.N.; Müller, D.B.; Liu, G. Elaborating the history of our cementing societies: An in-use stock perspective. *Environmental Science & Technology*, **2017**, *51* (19), 11468-11475.

(17) Gontia, P.; Nägeli, C.; Rosado, L.; Kalmykova, Y.; Österbring, M. Material-intensity database of residential buildings: A case-study of Sweden in the international context. *Resources, Conservation and Recycling*, **2018**, *130*, 228-239.

(18) Bergsdal, H., Brattebø, H.; Bohne, R. A.; Müller, D. B. Dynamic material flow analysis for Norway's dwelling stock. *Building Research & Information*, **2007**, *35* (5), 557-570.

(19) Müller, D.B. Stock dynamics for forecasting material flows—Case study for housing in The Netherlands. *Ecological Economics*, **2006**, *59* (1), 142-156.

(20) Pollard, J. H. A handbook of numerical and statistical techniques: With examples mainly from the life sciences. Cambridge University Press, Cambridge, 1977.

(21) Glöser, S.; Soulier, M.; Tercero Espinoza, L.A. Dynamic analysis of global copper flows. Global stocks, postconsumer material flows, recycling indicators, and uncertainty evaluation. *Environmental Science & Technology*, **2013**, *47* (12), 6564-6572.

(22) Cuéllar-Franca, R. M.; Azapagic, A. Environmental impacts of the UK residential sector: Life cycle assessment of houses. *Building and Environment*, **2012**, *54*, 86-99.

(23) Montgomery, D. C.; Peck, E. A.; Vining, G. G. *Introduction to linear regression analysis*. John Wiley & Sons, 2012.

(24) Fatima, T.; Xia, E.; Cao, Z.; Khan, D.; Fan, J.-L. Decomposition analysis of energy-related CO_2 emission in the industrial sector of China: evidence from the LMDI approach. *Environmental Science and Pollution Research*, **2019**, *26* (21), 21736-21749.

(25) Branger, F.; Quirion, P. Reaping the carbon rent: Abatement and overallocation profits in the European cement industry, insights from an LMDI decomposition analysis. *Energy Economics*, **2015**, *47*, 189-205.

(26) Feng, C.; Wang, M. Analysis of energy efficiency and energy savings potential in China's provincial industrial sectors. *Journal of Cleaner Production*, **2017**, *164*, 1531-1541.

(27) Lam, K. L.; Kenway, S. J.; Lane, J. L.; Islam, K. N.; de Berc, R. B. Energy intensity and embodied energy flow in Australia: An input-output analysis. *Journal of Cleaner Production*, **2019**, *226*, 357-368.

(28) Money Marks & Media. *'Building' up the GDP*, 2019. <http://j469.ascjclass.org/2019/09/03/building-up-the-gdp/> (accessed: 26 December 2020).

(29) Chen, W.-Q.; Graedel, T. E. In-use product stocks link manufactured capital to natural capital. *Proceedings of the National Academy of Sciences U.S.A.*, **2015**, *112* (20), 6265-6270.
